# A novel generalized Weibull Poisson G class of continuous probabilistic distributions with some copulas, properties and applications to real-life datasets

**DOI:** 10.1038/s41598-023-49873-w

**Published:** 2024-01-19

**Authors:** Atef F. Hashem, M. A. Abdelkawy, Abdisalam Hassan Muse, Haitham M. Yousof

**Affiliations:** 1 Department of Mathematics and Statistics, College of Science, Imam Mohammad Ibn Saud Islamic University (IMSIU), 11432 Riyadh, Saudi Arabia; 2 Mathematics and Computer Science Department, Faculty of Science, Beni-Suef University, Beni Suef, Egypt; 3 Faculty of Science and Humanities, School of Postgraduate Studies and Research (SPGSR), Amoud University, Borama, 25263 Somalia; 4 Department of Statistics, Mathematics and Insurance, Benha University, Benha, 13511 Egypt

**Keywords:** Statistics, Computational methods

## Abstract

The current study introduces and examines copula-coupled probability distributions. It explains their mathematical features and shows how they work with real datasets. Researchers, statisticians, and practitioners can use this study’s findings to build models that capture complex multivariate data interactions for informed decision-making. The versatility of compound G families of continuous probability models allows them to mimic a wide range of events. These incidents can range from system failure duration to transaction losses to annual accident rates. Due to their versatility, compound families of continuous probability distributions are advantageous. They can simulate many events, even some not well represented by other probability distributions. Additionally, these compound families are easy to use. These compound families can also show random variable interdependencies. This work focuses on the construction and analysis of the novel generalized Weibull Poisson-G family. Combining the zero-truncated-Poisson G family and the generalized Weibull G family creates the compound G family. This family’s statistics are mathematically analysed. This study uses Clayton, Archimedean-Ali-Mikhail-Haq, Renyi’s entropy, Farlie, Gumbel, Morgenstern, and their modified variations spanning four minor types to design new bivariate type G families. The single-parameter Lomax model is highlighted. Two practical examples demonstrate the importance of the new family.

## Introduction

The compound generators of probability distributions are widely employed in modeling a wide variety of data types, spanning fields such as reliability, insurance, reinsurance, economy, and engineering, among others. These versatile G families offer adaptable frameworks for understanding and analyzing complex phenomena in these domains. By combining multiple probability models, these compound families have the capacity to represent a diverse range of patterns and characteristics observed in real life data. In the context of reliability analysis, the modeling of failure times or durations often requires the use of compound distributions. These continuous compound distributions allow for the incorporation of various failure mechanisms and sources of uncertainty, including the failure of certain components, basic environmental factors, and maintenance (reliability) effects, to name a few. These examples illustrate the broad scope of failure mechanisms and sources of uncertainty that can be accommodated. The flexibility inherent in compound probability distribution families enables the effective capture of attributes such as heavy-tailedness (right and left), over-dispersion, and zero-inflation, which are commonly encountered in insurance-related data.

In the field of reliability analysis and engineering, compound probability G families serve as valuable tools for replicating different types of data, including data related to longevity, strength, degradation, and more. These distributions make it easier to investigate the many underlying mechanisms that combine to produce the paterns in the data that are seen.

This research endeavor aims to investigate and analyze a novel class of continuous probability distributions referred to as the G class. This exploration involves the use of a truncation process and its associated Poisson model under the zero-truncation (ZTP). The newly introduced set of continuous probabilistic possesses a strong rationale in both mathematical and statistical modeling contexts. Specifically, this study focuses on the Weibull-G (GW-G) distribution, which was recently proposed and examined by Cordeiro et al.^1^. The following equation represents the relevant cumulative distribution function (CDF) of the GW-G class:

1 $${\mathcal{G}}_{{\mathcalligra{b}\,\,}\,\,,{\mathcalligra{c}}\,,{\underline{\varvec{\mathcal{V}}}}}\left({\mathcalligra{w}}\,\right)={\left\{1-{\text{exp}}\left[-{\varvec{\mathcal{U}}}_{{\mathcalligra{c}}\,,{\underline{\varvec{\mathcal{V}}}}}\left({\mathcalligra{w}}\,\right)\right]\right\}}^{\mathcalligra{b}\,\,}\,\,\,\,{|}_{{\mathcalligra{w}}\,\in {\varvec{\mathcal{R}}}},$$

where the function ${\varvec{\mathcal{U}}}_{{\mathcalligra{c}}\,,{\underline{\varvec{\mathcal{V}}}}}\left({\mathcalligra{w}}\,\right)={\left[{\varvec{\mathcal{H}}}_{\underline{\varvec{\mathcal{V}}}}\left({\mathcalligra{w}}\,\right)/{{\overline{\varvec{\mathcal{H}}}}_{\underline{\varvec{\mathcal{V}}}}}\left({\mathcalligra{w}}\,\right)\right]}^{{\mathcalligra{c}}\,}{|}_{{\mathcalligra{w}}\,\in {\mathcal{R}}},$ is the odd-ratio function (ORF), the function ${{\varvec{\mathcal{H}}}_{\underline{\varvec{\mathcal{V}}}}}\left({\mathcalligra{w}}\,\right)$ represents the CDF of any selected base-line model with $\underline{\varvec{\mathcal{V}}}$ where $\underline{\varvec{\mathcal{V}}}$ is the parameters vector, the function ${{\overline{\varvec{\mathcal{H}}}}_{\underline{\varvec{\mathcal{V}}}}}\left({\mathcalligra{w}}\,\right)$ is the survival/reliability function (SF) base-line model, noting that $d{{\varvec{\mathcal{H}}}_{\underline{\varvec{\mathcal{V}}}}}\left({\mathcalligra{w}}\,\right)/d{\mathcalligra{w}}\,$ =${{\varvec{\mathcalligra{h}\,}}_{{\underline{\varvec{\mathcal{V}}}}}}\left({\mathcalligra{w}}\,\right)$ is the corresponding base-line PDF and $\mathcalligra{b}\,\,,{\mathcalligra{c}}\,>0$ are additional shape parameters. Staying in (1) and for ${\mathcalligra{c}}\,=2$, the GW-G family will reduce to simple generated-Rayleigh G (GR-G) (see Yousof et al.^2^ for more details and applications). Let the continuous RV ${Z}_{{\,\mathcalligra{u}\,\,}}$ denote the failure time of the ith minor component and let

$$W={\text{min}}\{{Z}_{1},{Z}_{2},\dots ,{Z}_{N}\}.$$

Then, the conditional CDF of $W$ given $N$ is

2 $$F\left({\mathcalligra{w}}\,{|}_{N}\right)=1-{\text{Pr}}\left(W>{\mathcalligra{w}}\,{|}_{N}\right)=1-{\left[1-{{\mathcal{G}}_{\mathcalligra{b}\,\,,{\mathcalligra{c}}\,,{\underline{\varvec{\mathcal{V}}}}}}\left({\mathcalligra{w}}\,\right)\right]}^{N}.$$

The determination of the unconditional cumulative distribution function (Un-CDF) for the generated Poisson Weibull-G (GPW-G) can be inferred and established using well-documented references, such as Alizadeh et al.^3^, Ramos et al.^4^, Korkmaz et al.^5^, Aryal and Yousof^6^, Abouelmagd et al.^7^, and Yousof et al.^8,9^, Al-Essa et al.^10^, Hamedani et al.^11^ and Salem et al.^12^ among others. Substituting Eq. (1) into Eq. (2) enables the derivation of the CDF for the GPW-G family as follows:

3 $${F}_{\underline{\varvec{\mathcal{P}}}}\left({\mathcalligra{w}}\,\right)={\nabla }_{{\mathcalligra{a}}\,}^{-1}\left[1-{\text{exp}}\left(-{\mathcalligra{a}}\,{\left\{1-{\text{exp}}\left[-{\varvec{\mathcal{U}}}_{{\mathcalligra{c}}\,,{\underline{\varvec{\mathcal{V}}}}}\left({\mathcalligra{w}}\,\right)\right]\right\}}^{\mathcalligra{b}\,\,}\,\,\,\,\right)\right]{|}_{{\mathcalligra{w}}\,\in {\varvec{\mathcal{R}}}},$$

where ${\underline{\varvec{\mathcal{P}}}}={\left({\mathcalligra{a}}\,,\mathcalligra{b}\,\,,{\mathcalligra{c}}\,,{\underline{\varvec{\mathcal{V}}}}^{{\text{T}}}\right)}^{{\text{T}}}$ is the compound parameters vector of the GPW-G family with already concludes another parameters vector for the base-line model and ${\nabla }_{{\mathcalligra{a}}\,}=1-{\text{exp}}\left(-{\mathcalligra{a}}\,\right)$. Therefore, the probability density function (PDF) of the GPW-G family reduces to

4 $${{f}_{\underline{\varvec{\mathcal{P}}}}}\left({\mathcalligra{w}}\,\right)={\mathcalligra{a}}\,{\mathcalligra{c}}\,\mathcalligra{b}\,\,{\nabla }_{{\mathcalligra{a}}\,}^{-1}{\text{exp}}\left(-{\mathcalligra{a}}\,{\left\{1-{\text{exp}}\left[-{\varvec{\mathcal{U}}}_{{\mathcalligra{c}}\,,{\underline{\varvec{\mathcal{V}}}}}\left({\mathcalligra{w}}\,\right)\right]\right\}}^{\mathcalligra{b}\,\,}\right)\frac{{{{\varvec{\mathcalligra{h}\,}}}\,\,_{\underline{\varvec{\mathcal{V}}}}}\left({\mathcalligra{w}}\,\right){{\varvec{\mathcal{H}}}_{\underline{\varvec{\mathcal{V}}}}}{\left({\mathcalligra{w}}\,\right)}^{{\mathcalligra{c}}\,-1}{\text{exp}}\left[-{\varvec{\mathcal{U}}}_{{\mathcalligra{c}}\,,{\underline{\varvec{\mathcal{V}}}}}\left({\mathcalligra{w}}\,\right)\right]}{{{\overline{\varvec{\mathcal{H}}}}_{\underline{\varvec{\mathcal{V}}}}}{\left({\mathcalligra{w}}\,\right)}^{{\mathcalligra{c}}\,+1}{\left\{1-{\text{exp}}\left[-{\varvec{\mathcal{U}}}_{{\mathcalligra{c}}\,,{\underline{\varvec{\mathcal{V}}}}}\left({\mathcalligra{w}}\,\right)\right]\right\}}^{1-\mathcalligra{b}\,\,}},$$

where ${\mathcalligra{w}}\,\in {\varvec{\mathcal{R}}}$. A RV $W$ having the PDF presented in Eq. (4) is denoted by $W\sim$ GPW-G $\left({\underline{\varvec{\mathcal{P}}}}\right)|{\underline{\varvec{\mathcal{P}}}}={\left({\mathcalligra{a}}\,,\mathcalligra{b}\,\,,{\mathcalligra{c}}\,,{\underline{\varvec{\mathcal{V}}}}^{{\text{T}}}\right)}^{{\text{T}}}$. Below, some special models from the GPW-G class, see Table 1 which contains six special models.

**Table 1:** Special cases from the GPW-G generator.

No.	${\mathcalligra{a}}\,$	$\mathcalligra{b}\,\,$	${\mathcalligra{c}}\,$	Quasi (pseudo) model	Quasi (pseudo) CDF
1	1	$\mathcalligra{b}\,\,$	${\mathcalligra{c}}\,$	Pseudo/Quasi-GPW-G|$\mathcalligra{w} \in \text {R}$	$\left[1-{\text{exp}}\left(-{\left\{1-{\text{exp}}\left[-{{\varvec{\mathcal{U}}}_{{\mathcalligra{c}}\,,{\underline{\varvec{\mathcal{V}}}}}}\left({\mathcalligra{w}}\,\right)\right]\right\}}^{\mathcalligra{b}\,\,}\,\,\,\,\right)\right]{\nabla }_{1}^{-1}$
2	1	1	${\mathcalligra{c}}\,$	Pseudo/Quasi-PW-G|$\mathcalligra{w} \in \text {R}$	$\left[1-{\text{exp}}\left(-\left\{1-{\text{exp}}\left[-{{\varvec{\mathcal{U}}}_{{\mathcalligra{c}}\,,{\underline{\varvec{\mathcal{V}}}}}}\left({\mathcalligra{w}}\,\right)\right]\right\}\right)\right]{\nabla }_{1}^{-1}$
3	1	$\mathcalligra{b}\,\,$	1	Pseudo/Quasi-PGE-G|$\mathcalligra{w} \in \text {R}$	$\left[1-{\text{exp}}\left(-{\left\{1-{\text{exp}}\left[-{{\varvec{\mathcal{U}}}_{1,{\underline{\varvec{\mathcal{V}}}}}}\left({\mathcalligra{w}}\,\right)\right]\right\}}^{\mathcalligra{b}\,\,}\,\,\,\,\right)\right]{{\nabla }_{1}^{-1}}$
4	1	$\mathcalligra{b}\,\,$	2	Pseudo/Quasi-PBX-G|$\mathcalligra{w} \in \text {R}$	$\left[1-{\text{exp}}\left(-{\left\{1-{\text{exp}}\left[-{{\varvec{\mathcal{U}}}_{{2,{\underline{\varvec{\mathcal{V}}}}}}}\left({\mathcalligra{w}}\,\right)\right]\right\}}^{\mathcalligra{b}\,\,}\,\,\,\,\right)\right]{\nabla }_{1}^{-1}$
5	${\mathcalligra{a}}\,$	$\mathcalligra{b}\,\,$	2	BX-G|$\mathcalligra{w} \in \text {R}$	$\left[1-{\text{exp}}\left(-{\mathcalligra{a}}\,{\left\{1-{\text{exp}}\left[-{\varvec{\mathcal{U}}}_{{2,{\underline{\varvec{\mathcal{V}}}}}}\left({\mathcalligra{w}}\,\right)\right]\right\}}^{\mathcalligra{b}\,\,}\,\,\,\,\right)\right]{\nabla }_{{\mathcalligra{a}}\,}^{-1}$
6	${\mathcalligra{a}}\,$	1	1	PG-G|$\mathcalligra{w} \in \text {R}$	$\left[1-{\text{exp}}\left(-{\mathcalligra{a}}\,\left\{1-{\text{exp}}\left[-{{\varvec{\mathcal{U}}}_{{1,{\underline{\varvec{\mathcal{V}}}}}}}\left({\mathcalligra{w}}\,\right)\right]\right\}\right)\right]{\nabla }_{{\mathcalligra{a}}\,}^{-1}$

Utilizing established copula models such as the Farlie copula, Gumbel, copula and Morgenstern copula (FRGM), its modified form, the Clayton copula (CTN), and Renyi copula (under the entropy concept), novel bivariate and multivariate GPW-G families are formulated and analyses. As articulated by Fisher^13^, statisticians are drawn to copulas primarily for two key purposes: firstly, to assess scale-independent measures of dependence, and secondly, as a foundational framework for obtaining and analysing new bivariate (and multivariate) G families of probabilistic distribution. Fisher underscored these dual motivations as central to the interest in copulas. Specifically, copulas play a pivotal role in examining the interplay between two variables, as they enable the separation of the influences of marginal distributions from the effects of interdependence, thus serving as a fundamental tool in the analysis of dependence.

Copulas play a significant role in modeling dependencies between random variables. The inclusion of copulas in the study allows for the exploration of how variables within this new distribution family are interrelated. This is essential for understanding and modeling multivariate data, which is common in many real life applications. The paper applies the newly introduced GPW-G family and copulas to real-life datasets. This step is essential to demonstrate the practical relevance and usefulness of the proposed statistical framework. It shows how these mathematical concepts can be applied to solve real life problems, which is a fundamental aspect of statistics. The development of new families of probability distributions contributes to the advancement of statistical science. It opens up opportunities for innovative research and applications across various domains, including finance, engineering, health, and more. Statistical research often involves comparing different models and distributions to identify the most suitable one for a given dataset. The paper’s exploration of various goodness-of-fit statistic tests, such as Akaike-Information-Criterion and Bayesian-Information-Criterion, helps researchers evaluate the performance of the new distribution family compared to existing models. In summary, the statistical motivations for introducing this paper revolve around the need for more flexible, tailored, and innovative probability distribution families that can accurately capture complex data patterns and dependencies. The paper aims to contribute to the field of statistics by providing a comprehensive understanding of the proposed distribution family’s mathematical properties and demonstrating its practical utility through real-life applications.

The use of the generalized Weibull Poisson G family will be appropriate when we are dealing with data that does not have outliers. It may also be useful to use the new family model when modeling data that has an increased failure rate. It may be appropriate to use the new family when we are dealing with data twisted to the right, whether it has a heavy tail or a light tail. The use of the GPW-G family will be appropriate when we are dealing with data which have decreasing-constant hazard rate function, data which have the increasing hazard rate function, data which have the increasing-constant-decreasing hazard rate function or the U- hazard rate function and finally data which have the constant hazard rate function.

The bivariate GPW-G families introduced in this publication offer promising avenues for future research, warranting dedicated investigations (refer to “Bivariate versions” section). The “Main mathematical properties” section of this work presents and scrutinizes pertinent mathematical and statistical characteristics. “Estimation” and “Special case” sections delve into the well-established maximum probability method and its practical applications in real life scenarios. Concluding remarks are provided in the “Simulation study” section.

## Bivariate versions

A copula stands as a pivotal concept within the realm of statistics, serving as a fundamental tool for characterizing information sets encompassing two or more variables. It functions as a mathematical construct establishing the relationship between the marginal distributions of multiple variables and their collective joint distribution. Notably, the marginal distributions can exhibit both independence and interdependence. In recent years, copulas have experienced a surge in prominence due to their versatility and ability to model intricate patterns of interdependency among variables. This surge is attributed to their capacity to effectively represent complex dependency structures. The significance and applications of copulas in statistical modeling and bivariate data analysis are exemplified in the following ways:

I. Copulas find application in illustrating the reciprocal dependence of variables, a fundamental concept across various subfields within data and statistical research. By employing copulas, one can accurately depict the joint PDFs of all variables while preserving the integrity of their marginal models. This feature enables the capture of intricate relationships between variables, which can be challenging to quantify using conventional correlation metrics.
II. In the realm of risk assessment and analysis and financial studies, copulas are commonly used to describe the statistical relationship between the expected returns on some assets. This financial process is very crucial for the optimization of the portfolio returns, where the goal is to construct portfolios that maximize returns while minimizing risk. Copulas facilitate a more precise risk analysis of portfolio components, aiding in the creation of more effective investment portfolios.
III. Copulas play a vital role in risk analysis, risk management, reinsurance and insurance by modeling the dependence structures between variables. This modeling enhances the assessment of the probability of expected/catastrophic events, such as market crashes or natural disasters, providing a more accurate estimation of their likelihood.
IV. Copulas serve as a valuable tool for data generation, particularly in simulation experiments and studies where obtaining/collecting real-life data sets is challenging or costly. They enable the simulation of new data sets with the same dependence structures as the original data, facilitating testing and analysis. To achieve this, copulas are employed to model the dependence structure among variables.

### BGPW-G type via FRGM copula

Exploring the interconnectedness among multiple random variables is effectively achieved by employing a parametric copula family known as the FRGM copula. This copula serves as an initial approximation to well-established copulas like Ali-Mikhail-Haq, Frank, and other copulas. What distinguishes it is its straightforward formulation, which facilitates explicit calculus, resulting in precise outcomes (as extensively detailed in Ref.^14^). The FRGM copula’s ability to mimic a wide range of dependency structures—including mixed, positive, and negative dependencies—adds to its allure. Because of its versatility, it is now necessary in many areas, including financial risk management, insurance risk management, and environmental risk assessment. The FRGM copula, for instance, has been useful in illustrating relationships between environmental variables, insurance claims interdependencies, and asset values.

The FRGM copula emerges as a flexible tool, ideally suited for capturing a diverse array of dependency structures and primarily geared toward simulating random variable behavior. Its user-friendliness and advantages, such as the capacity to represent mixed dependencies, distinguish it from other copula families. In this section, we introduce innovative bivariate GPW-G (Bv-GPW-G) variants by leveraging the FRGM copula. This copula’s lineage can be traced back to contributions by Morgenstern^15^, Farlie^16^, Gumbel^17,18^, Johnson and Kotz^19^, Balakrishnan and Lai^20^, and Johnson and Kotz^21^. Additionally, we consider the ‘modified FRGM (M-FRGM)’ copula, the ‘CTN copula’, the ‘Archimedean-Ali-Mikhail-Haq’ copula, and the ‘Renyi’s entropy’ copula (for more detailed insights, consult^14,20,22^. Furthermore, we present the Multivariate GPW-G (Mv GPW-G) type (for further clarification, please refer to Ref.^20^. Finally, we outline potential avenues for future research dedicated to investigating and analyzing these novel models.

This section offers a comprehensive exploration of copulas, encompassing their fundamental role in modeling interdependencies among variables. A thorough examination of copulas delves into their mathematical intricacies, including attributes such as tail dependency, association measures, and transformation properties. Various copula families, including Archimedean, elliptical, and vine copulas, are scrutinized, each characterized by its unique properties.

Furthermore, the FRGM copula finds noteworthy applications across diverse domains, exemplified as follows:

I. The FRGM copula serves as a valuable tool for constructing models that elucidate the interdependencies among asset prices. These models are instrumental in devising hedging strategies and effectively assessing portfolio risk.
II. In the realm of insurance, the FRGM copula is employed to model the dependence between insurance claims. This modeling aids in gauging the level of risk faced by insurance firms, facilitating the formulation of pricing and reserving strategies.
III. The FRGM copula plays a pivotal role in constructing models that capture the dependencies among environmental variables. These models are instrumental in evaluating the likelihood of ecological catastrophes and devising strategies for mitigating their impacts.

These applications underscore the versatility and practical relevance of the FRGM copula in addressing complex problems across various fields.

 Let us start with the common joint-CDF of the FRGM type, where $\Phi \in \left[-\mathrm{1,1}\right]$ is the parameter which controls the dependance, we have

5 $${\mathbb{F}}_{\Phi }({\mathbb{T}},{\mathbb{S}})={\mathbb{T}}{\mathbb{S}}+\Phi {\mathbb{T}}\overline{\mathbb{S}}{\mathbb{S}}\overline{\mathbb{T}},$$

where ${\mathbb{T}}={\mathbb{T}}(.)\in (\mathrm{0,1})$, ${\mathbb{S}}={\mathbb{S}}(.)\in (\mathrm{0,1})$, are two continuous functions. Then, based on (5) we have the following main results:

$${\mathbb{F}}_{\Phi }({\mathbb{T}},0)={\mathbb{F}}_{\Phi }(0,{\mathbb{S}})=0|{\mathbb{T}}={\mathbb{T}}\left(.\right)\in \left(\mathrm{0,1}\right),{\mathbb{S}}={\mathbb{S}}\left(.\right)\in \left(\mathrm{0,1}\right),$$

which refers to the “grounded-minimum condition” and ${\mathbb{F}}_{\Phi }({\mathbb{T}},1)={\mathbb{T}}$ and ${\mathbb{F}}_{\Phi }(1,{\mathbb{S}})={\mathbb{S}}$ which, analogusely, refers to the “grounded-maximum condition”. Then, setting

6 $${\overline{\mathbb{T}}}={\overline{\mathbb{T}}}_{{\underline{\varvec{\mathcal{P}}}}_{1}}=1-{{\nabla }_{{\mathcalligra{a}}\,_{1}}^{-1}}\left[1-{\text{exp}}\left(-{{\mathcalligra{a}}\,_{1}}{\mathcal{G}}_{{{\mathcalligra{b}\,\,}\,\,_{1}},{{\mathcalligra{c}}\,_{1}},{\underline{\varvec{\mathcal{V}}}}}\left({\mathcalligra{w}}\,_{1}\right)\right)\right]|{\underline{\varvec{\mathcal{P}}}}_{1}>0,$$

where ${{\mathcal{G}}_{{\mathcalligra{b}\,\,}\,\,_{\,\mathcalligra{u}\,\,}},{{\mathcalligra{c}}\,_{\,\mathcalligra{u}\,\,}},{\underline{\varvec{\mathcal{V}}}}}\left({{\mathcalligra{w}}\,}_{1}\right)$ is the CDF corresponding to ${\mathcal{G}}_{{\mathcalligra{b}\,\,}\,\,,{\mathcalligra{c}}\,,{\underline{\varvec{\mathcal{V}}}}}\left({\mathcalligra{w}}\,\right)$ defined in (1) and

7 $$\overline{\mathbb{S}}={\overline{\mathbb{S}}}_{{{\underline{\varvec{\mathcal{P}}}}}_{2}}=1-{\nabla }_{{{\mathcalligra{a}}\,}_{2}}^{-1}\left[1-{\text{exp}}\left(-{{\mathcalligra{a}}\,}_{2}{\mathcal{G}}_{{{\mathcalligra{b}\,\,}\,\,_{2}},{{\mathcalligra{c}}\,_{2}},{\underline{\varvec{\mathcal{V}}}}}\left({{\mathcalligra{w}}\,}_{2}\right)\right)\right]|{{\underline{\varvec{\mathcal{P}}}}}_{2}>0.$$

Then, using Eqs. (6) and (7) we get

8 $$\begin{aligned}F({{\mathcalligra{w}}\,}_{1},{{\mathcalligra{w}}\,}_{2})&={\mathbb{F}}({F}_{{{\underline{\varvec{\mathcal{P}}}}}_{1}}\left({{\mathcalligra{w}}\,}_{1}\right),{F}_{{{\underline{\varvec{\mathcal{P}}}}}_{2}}\left({{\mathcalligra{w}}\,}_{2}\right))\\ &={\nabla }_{{{\mathcalligra{a}}\,}_{1}}^{-1}{\nabla }_{{{\mathcalligra{a}}\,}_{2}}^{-1}\left[1-{\text{exp}}\left(-{{\mathcalligra{a}}\,}_{1}{\mathcal{G}}_{{\mathcalligra{b}\,\,}\,\,_{1},{{\mathcalligra{c}}\,}_{1},{\underline{\varvec{\mathcal{V}}}}}\left({{\mathcalligra{w}}\,}_{1}\right)\right)\right]\times \left[1-{\exp}\left(-{{\mathcalligra{a}}\,}_{2}{\mathcal{G}}_{{\mathcalligra{b}\,\,}\,\,_{2},{{\mathcalligra{c}}\,}_{{2},{\underline{\varvec{\mathcal{V}}}}}}\left({{\mathcalligra{w}}\,}_{2}\right)\right)\right]\\ &\quad\times \left[1+\Phi \left(\begin{array}{c}\left\{1-{\nabla }_{{{\mathcalligra{a}}\,}_{1}}^{-1}\left[1-{\exp}\left(-{{\mathcalligra{a}}\,}_{1}{\mathcal{G}}_{{\mathcalligra{b}\,\,}\,\,_{1},{{\mathcalligra{c}}\,}_{{1},{\underline{\varvec{\mathcal{V}}}}}}\left({{\mathcalligra{w}}\,}_{1}\right)\right)\right]\right\}\\ \left\{1-{\nabla }_{{{\mathcalligra{a}}\,}_{2}}^{-1}\left[1-{\exp}\left(-{{\mathcalligra{a}}\,}_{2}{\mathcal{G}}_{{\mathcalligra{b}\,\,}\,\,_{2},{{\mathcalligra{c}}\,}_{{2},{\underline{\varvec{\mathcal{V}}}}}}\left({{\mathcalligra{w}}\,}_{2}\right)\right)\right]\right\}\end{array}\right)\right].\end{aligned}$$

Equation (8) makes it simple to calculate the equivalent PDF for the bivariate data sets, which can then be utilized in statistical modeling.

### BGPW-G type via M-FRGM copula

The M-FRGM copula can be considered as a flexible variation (version) of the original FRGM copula, providing the capability to capture a wide range of dependency patterns. It stands out due to its ease of usage and many other several advantages compared to other copula G families, particularly its ability to model mixed dependencies (for more in-depth information, refer to Ref.^23^. As a result, the FRGM copula is widely favored and applied in a diverse range of applications.

In the following subsection, we explore a specific variant of this copula. The Copula of the M-FRGM can be mathematically re-expressed by the following formula:

9 $${\mathbb{F}}\left({\mathbb{T}},{\mathbb{S}},\Phi \right)=\Phi \widetilde{{\varvec{G}}\left({\mathbb{T}}\right)}\widetilde{{\varvec{B}}\left({\mathbb{S}}\right)}+{\mathbb{T}}{\mathbb{S}},$$

where $\widetilde{{\varvec{G}}({\mathbb{T}})}={\mathbb{T}}\overline{{\varvec{G}}({\mathbb{T}})}$, $\widetilde{{\varvec{B}}\left({\mathbb{S}}\right)}={\mathbb{S}}\overline{{\varvec{B}}\left({\mathbb{S}}\right)}$ and ${\varvec{G}}\left({\mathbb{T}}\right)$ and ${\varvec{B}}\left({\mathbb{S}}\right)$ are two absolutely continuous functions on $(\mathrm{0,1})$ with the following condition boundary condition:

$$0={\varvec{G}}\left(0\right)={\varvec{B}}\left(0\right)={\varvec{G}}\left(1\right)={\varvec{B}}\left(1\right),$$

where

10 $$K({\mathbb{T}})=inf\left\{\frac{\partial }{\partial {\mathbb{T}}}\widetilde{{\varvec{G}}({\mathbb{T}})}:{{\mathcalligra{h}\,}}_{1}({\mathbb{T}})\right\}<0,L({\mathbb{T}})=sup\left\{\frac{\partial }{\partial {\mathbb{T}}}\widetilde{{\varvec{G}}({\mathbb{T}})}:{{\mathcalligra{h}\,}}_{1}({\mathbb{T}})\right\}<0,$$

11 $$k\left({\mathbb{S}}\right)=inf\left\{\frac{\partial }{\partial {\mathbb{S}}}\widetilde{{\varvec{B}}\left({\mathbb{S}}\right)}:{{\mathcalligra{h}\,}}_{2}\left({\mathbb{S}}\right)\right\}>0,l\left({\mathbb{S}}\right)=sup\left\{\frac{\partial }{\partial {\mathbb{S}}}\widetilde{{\varvec{B}}\left({\mathbb{S}}\right)}:{{\mathcalligra{h}\,}}_{2}\left({\mathbb{S}}\right)\right\}>0.$$

Then,

$$\min\left(K({\mathbb{T}})L({\mathbb{T}}),k\left({\mathbb{S}}\right)l\left({\mathbb{S}}\right)\right)\ge 1,$$

where

12 $$\frac{\partial }{\partial {\mathbb{T}}}\widetilde{{\varvec{G}}({\mathbb{T}})}={\varvec{G}}\left({\mathbb{T}}\right)+{\mathbb{T}}\frac{\partial }{\partial {\mathbb{T}}}{\varvec{G}}\left({\mathbb{T}}\right),$$

13 $${{\mathcalligra{h}\,}}_{1}({\mathbb{T}})=\left\{{\mathbb{T}}:{\mathbb{T}}\in \left(\mathrm{0,1}\right)|\frac{\partial }{\partial {\mathbb{T}}}\widetilde{{\varvec{G}}({\mathbb{T}})}\text{ exists}\right\}, {{\mathcalligra{h}\,}}_{2}\left({\mathbb{S}}\right)=\left\{{\mathbb{S}}:{\mathbb{S}}\in \left(\mathrm{0,1}\right)|\frac{\partial }{\partial {\mathbb{S}}}\widetilde{{\varvec{B}}\left({\mathbb{S}}\right)}\text{ exists}\right\}.$$

The versatility of the M-FRGM copula finds practical applications in various scenarios, as illustrated below:

I. Using the M-FRGM copula, one can simulate and analyze the relationship between asset prices. This capability is valuable for devising strategies to hedge risks and assessing the risk associated with asset portfolios.
II. The M-FRGM copula can be applied to construct models that portray the connections among environmental variables. These models allow for the assessment of the probability of environmental disasters and the creation of strategies for prevention and mitigation.
III. The M-FRGM copula serves as a representation of the interconnections among insurance claims. This representation assists in quantifying the level of risk exposure for insurance firms and facilitates the development of pricing and reserving policies.
IV. In the current scope of this work, we will embark on a theoretical exploration of four distinct forms of the M-FRGM copula, delving into their mathematical properties and implications.

#### The type I M-FRGM copula

Think about the two practical versions ${\varvec{G}}({\mathbb{T}})$ and ${\varvec{B}}\left({\mathbb{S}}\right)$, then the Type I of the BGPW-G class according to the M-FRGM copula can then be expressed as

14 $${\mathbb{F}}_{\Phi }({\mathbb{T}},{\mathbb{S}})=\Phi \left[\widetilde{{\varvec{G}}({\mathbb{T}})}\widetilde{{\varvec{B}}\left({\mathbb{S}}\right)} \right]+\left(\begin{array}{c}\left\{{\nabla }_{{{\mathcalligra{a}}\,}_{1}}^{-1}\left[1-{\text{exp}}\left(-{{\mathcalligra{a}}\,}_{1}{\mathcal{G}}_{{\mathcalligra{b}\,\,}\,\,_{1},{{\mathcalligra{c}}\,}_{{1},{\underline{\varvec{\mathcal{V}}}}}}\left({\mathbb{T}}\right)\right)\right]\right\}\\ \times \left\{{\nabla }_{{{\mathcalligra{a}}\,}_{2}}^{-1}\left[1-{\text{exp}}\left(-{{\mathcalligra{a}}\,}_{2}{\mathcal{G}}_{{\mathcalligra{b}\,\,}\,\,_{2},{{\mathcalligra{c}}\,}_{{2},{\underline{\varvec{\mathcal{V}}}}}}\left({\mathbb{S}}\right)\right)\right]\right\}\end{array}\right),$$

where

$$\widetilde{{\varvec{G}}({\mathbb{T}})}={\mathbb{T}}\left\{{1-\nabla }_{{{\mathcalligra{a}}\,}_{1}}^{-1}\left[1-{\text{exp}}\left(-{{\mathcalligra{a}}\,}_{1}{\mathcal{G}}_{{\mathcalligra{b}\,\,}\,\,_{1},{{\mathcalligra{c}}\,}_{{1},{\underline{\varvec{\mathcal{V}}}}}}\left({\mathbb{T}}\right)\right)\right]\right\}|{{\underline{\varvec{\mathcal{P}}}}}_{1}>0,$$

and

$$\widetilde{{\varvec{B}}\left({\mathbb{S}}\right)}={\mathbb{S}}\left\{1-{\nabla }_{{{\mathcalligra{a}}\,}_{2}}^{-1}\left[1-{\text{exp}}\left(-{{\mathcalligra{a}}\,}_{2}{\mathcal{G}}_{{\mathcalligra{b}\,\,}\,\,_{2},{{\mathcalligra{c}}\,}_{{2},{\underline{\varvec{\mathcal{V}}}}}}\left({\mathbb{S}}\right)\right)\right]\right\}|{{\underline{\varvec{\mathcal{P}}}}}_{2}>0.$$

Equation (14) refers to the CDF of the type I BGPW-G via the M-FRGM which can be easily employed for deriving the corresponding PDF. The new PDF of the type I due to the M-FRGM can be used in statistical modeling for the bivariate data sets.

#### The type II M-FRGM copula

Let ${\varvec{G}}({\mathbb{T}})$ and ${\varvec{B}}\left({\mathbb{S}}\right)$ be two continuous functions and

15 $${\varvec{G}}\left({\mathbb{T}}\right)|{\Phi }_{1}>0={\left(1-{\mathbb{T}}\right)}^{1-{\Phi }_{1}}{\mathbb{T}}^{{\Phi }_{1}}\text{ and }{\varvec{B}}\left({\mathbb{S}}\right)|{\Phi }_{2}>0={\left(1-{\mathbb{S}}\right)}^{1-{\Phi }_{2}}{\mathbb{S}}^{{\Phi }_{2}}.$$

Then, the BGPW-G version of the Type II can be written as

16 $${\mathbb{F}}_{\Phi ,{\Phi }_{1},{\Phi }_{2}}({\mathbb{T}},{\mathbb{S}})=\left[\Phi {\left(1-{\mathbb{S}}\right)}^{1-{\Phi }_{1}}{\left(1-{\mathbb{S}}\right)}^{1-{\Phi }_{2}}{\mathbb{T}}^{{\Phi }_{1}}{\mathbb{S}}^{{\Phi }_{2}}+1\right]{\mathbb{T}}{\mathbb{S}}.$$

#### The type III M-FRGM copula

Let

17 $$\widetilde{{\varvec{C}}\left({\mathbb{T}}\right)}={\mathbb{T}}\left[{\text{log}}\left(1+\overline{\mathbb{T}}\right)\right]\text{ and }\widetilde{{\varvec{D}}\left({\mathbb{S}}\right)}={\mathbb{S}}\left[{\text{log}}\left(1+\overline{\mathbb{S}}\right)\right].$$

Due to Ghosh and Ray^24^, we have

18 $${\mathbb{F}}_{\Phi }\left({\mathbb{T}},{\mathbb{S}}\right)={\mathbb{T}}{\mathbb{S}}\left[1+\Phi \widetilde{{\varvec{C}}\left({\mathbb{T}}\right)}\widetilde{{\varvec{D}}\left({\mathbb{S}}\right)}\right].$$

#### The type IV M-FRGM copula

Due to Ghosh and Ray, consider ${F}^{-1}\left({\mathbb{T}}\right)$ and ${F}^{-1}\left({\mathbb{S}}\right)$. Then

19 $${\mathbb{F}}({\mathbb{T}},{\mathbb{S}})={F}^{-1}({\mathbb{T}}){\mathbb{T}}+{F}^{-1}({\mathbb{S}}){\mathbb{S}}-{F}^{-1}({\mathbb{S}}){F}^{-1}({\mathbb{T}}),$$

where $${F}^{-1}\left({\mathbb{T}}\right)=Q\left({\mathbb{T}}\right)$$ and $${F}^{-1}\left({\mathbb{S}}\right)=Q\left({\mathbb{S}}\right).$$

### CTN copula

The CTN copula is versatile and lends itself to a range of interpretations, including but not limited to the following:

20 $${\mathbb{F}}\left({\mathbb{T}}_{1},{\mathbb{T}}_{2}\right)|\Phi \in \left[0,\infty \right]={\left({\mathbb{T}}_{1}^{-\Phi }-1+{\mathbb{T}}_{2}^{-\Phi }\right)}^{-\frac{1}{\Phi }}.$$

Let us assume that ${\mathbb{T}}\sim$ GPW-G $({{\mathcalligra{a}}\,}_{1},{\mathcalligra{b}\,\,}\,\,_{1},{{\mathcalligra{c}}\,}_{1})$ and $W\sim$ GPW-G $({{\mathcalligra{a}}\,}_{2},{\mathcalligra{b}\,\,}\,\,_{2},{{\mathcalligra{c}}\,}_{2})$. Then, setting

$${\mathbb{T}}_{1}={\mathbb{T}}\left(\mathcalligra{m}\right)={\nabla }_{{{\mathcalligra{a}}\,}_{1}}^{-1}\left[1-{\text{exp}}\left(-{{\mathcalligra{a}}\,}_{1}{\mathcal{G}}_{{\mathcalligra{b}\,\,}\,\,_{1},{{\mathcalligra{c}}\,}_{{1},{\underline{\varvec{\mathcal{V}}}}}}\left(\mathcalligra{m}\right)\right)\right]{|}_{{{\underline{\varvec{\mathcal{P}}}}}_{1}>0},$$

and

$${\mathbb{T}}_{2}={\mathbb{T}}\left({\mathcalligra{w}}\,\right)={\nabla }_{{{\mathcalligra{a}}\,}_{2}}^{-1}\left[1-{\text{exp}}\left(-{{\mathcalligra{a}}\,}_{2}{\mathcal{G}}_{{\mathcalligra{b}\,\,}\,\,_{2},{{\mathcalligra{c}}\,}_{{2},{\underline{\varvec{\mathcal{V}}}}}}\left({\mathcalligra{w}}\,\right)\right)\right]{|}_{{{\underline{\varvec{\mathcal{P}}}}}_{2}>0},$$

Then, based on (20) the new BGPW-G type statistical model using the CTN copula can then be addressed by

21 $${\mathbb{F}}(\mathcalligra{m},{\mathcalligra{w}}\,)={\mathbb{F}}({F}_{{{\underline{\varvec{\mathcal{P}}}}}_{1}}\left(\mathcalligra{m}\right),{F}_{{{\underline{\varvec{\mathcal{P}}}}}_{2}}\left({\mathcalligra{w}}\,\right))={\left[\begin{array}{c}{\left({\nabla }_{{{\mathcalligra{a}}\,}_{1}}^{-1}\left[1-{\text{exp}}\left(-{{\mathcalligra{a}}\,}_{1}{\mathcal{G}}_{{\mathcalligra{b}\,\,}\,\,_{1},{{\mathcalligra{c}}\,}_{{1},{\underline{\varvec{\mathcal{V}}}}}}\left(\mathcalligra{m}\right)\right)\right]\right)}^{-\Phi }\\ +{\left({\nabla }_{{{\mathcalligra{a}}\,}_{2}}^{-1}\left[1-{\text{exp}}\left(-{{\mathcalligra{a}}\,}_{2}{\mathcal{G}}_{{\mathcalligra{b}\,\,}\,\,_{2},{{\mathcalligra{c}}\,}_{{2},{\underline{\varvec{\mathcal{V}}}}}}\left({\mathcalligra{w}}\,\right)\right)\right]\right)}^{-\Phi }\\ -1\end{array}\right]}^{-\frac{1}{\Phi }}.$$

### Renyi’s entropy

The Renyi’s entropy copula offers a simplified approach to obtaining bivariate probability distributions, hence obviating the necessity for intricate mathematical derivations. The implementation of this simplified methodology improves the efficiency of mathematical and statistical modeling in the context of analyzing bivariate data. The derivation of the bivariate form of Renyi’s entropy copula can be achieved by utilizing two functions, T and S, which are intrinsic to Renyi’s entropy copula. Significantly, the integration of Renyi’s entropy copula into a novel bivariate model does not introduce any supplementary parameters. To gain a thorough understanding of Renyi’s entropy copula, a highly informative resource is the study conducted by Pougaza and Djafari^22^. Upon integrating this body of knowledge with the theories stated by Pougaza and Djafari^22^, a number of observations can be made. Then,

22 $${\mathbb{F}}({\mathbb{T}},{\mathbb{S}})={{\mathcalligra{w}}\,}_{2}{\mathbb{T}}-{{\mathcalligra{w}}\,}_{1}{{\mathcalligra{w}}\,}_{2}+{{\mathcalligra{w}}\,}_{1}{\mathbb{S}},$$

then, using (22) the associated CDF of the BGPW-G using the Renyi’s entropy can be derived form

23 $${\mathbb{F}}\left({{\mathcalligra{w}}\,}_{1},{{\mathcalligra{w}}\,}_{2}\right)=+{{\mathcalligra{w}}\,}_{2}\left\{{\nabla }_{{{\mathcalligra{a}}\,}_{1}}^{-1}\left[1-{\exp}\left(-{{\mathcalligra{a}}\,}_{1}{\mathcal{G}}_{{\mathcalligra{b}\,\,}\,\,_{1},{{\mathcalligra{c}}\,}_{{1},{\underline{\varvec{\mathcal{V}}}}}}\left(\mathcalligra{m}\right)\right)\right]\right\}-{{\mathcalligra{w}}\,}_{1}{{\mathcalligra{w}}\,}_{2}+{{\mathcalligra{w}}\,}_{1}\left\{{\nabla }_{{{\mathcalligra{a}}\,}_{2}}^{-1}\left[1-{\exp}\left(-{{\mathcalligra{a}}\,}_{2}{\mathcal{G}}_{{\mathcalligra{b}\,\,}\,\,_{2},{{\mathcalligra{c}}\,}_{{2},{\underline{\varvec{\mathcal{V}}}}}}\left({\mathcalligra{w}}\,\right)\right)\right]\right\}.$$

### Ali-Mikhail-Haq copula

Eliminating the need for complex mathematical derivations, the Ali-Mikhail-Haq (Archimedean type) copula makes the construction of bivariate probability distributions easier. The process of mathematically and statistically defining data with two variables is simplified as a result of this. According to Ali et al.^14^, one may use the two functions T and S to obtain the bivariate version of the Archimedean Ali-Mikhail-Haq copula. This can be done by following the instructions provided. When using the Archimedean Ali-Mikhail-Haq copula, the incorporation of new parameters into the recently established bivariate model is carried out in a step-by-step manner, with each new parameter being introduced one at a time. Balakrishnan and Lai^20^ and Ali et al.^14^ are two important sources that should be consulted while attempting to comprehend the Archimedean Ali-Mikhail-Haq copula. In addition, the following basic formula can be used as a guide to construct the unique joint CDF of the Archimedean Ali-Mikhail-Haq copula, guaranteeing the satisfaction of a more strict Lipschitz condition:

24 $${\mathbb{F}}\left({\mathbb{T}},{\mathbb{S}}\right)={\mathbb{T}}{\mathbb{S}}\frac{1}{1-\Phi \overline{\mathbb{T}}\overline{\mathbb{S}}}|\Phi \in \left(-\mathrm{1,1}\right).$$

Therefore, in order to find the corresponding joint density, the following formula needs to be utilized:

25 $${\mathcalligra{c}}\,\left({\mathbb{T}},{\mathbb{S}}\right)=\frac{1}{{\left[1-\Phi \overline{\mathbb{T}}\overline{\mathbb{S}}\right]}^{2}}\left(1-\Phi +2\Phi \frac{{\mathbb{T}}{\mathbb{S}}}{1-\Phi \overline{\mathbb{T}}\overline{\mathbb{S}}}\right)|\Phi \in \left(-\mathrm{1,1}\right).$$

Then, for any continuous RVs ${\mathbb{T}}\sim$ GPW-G $({{\mathcalligra{a}}\,}_{1},{\mathcalligra{b}\,\,}\,\,_{1},{{\mathcalligra{c}}\,}_{1})$ and $W\sim$ GPW-G $({{\mathcalligra{a}}\,}_{2},{\mathcalligra{b}\,\,}\,\,_{2},{{\mathcalligra{c}}\,}_{2})$ we arrive at

26 $${\mathbb{F}}\left({\mathbb{T}},{\mathbb{S}}\right)=\frac{{\nabla }_{{{\mathcalligra{a}}\,}_{1}}^{-1}\left[1-{\exp}\left(-{{\mathcalligra{a}}\,}_{1}{\mathcal{G}}_{{\mathcalligra{b}\,\,}\,\,_{1},{{\mathcalligra{c}}\,}_{{1},{\underline{\varvec{\mathcal{V}}}}}}\left(\mathcalligra{m}\right)\right)\right]{\nabla }_{{{\mathcalligra{a}}\,}_{2}}^{-1}\left[1-{\text{exp}}\left(-{{\mathcalligra{a}}\,}_{2}{\mathcal{G}}_{{\mathcalligra{b}\,\,}\,\,_{2},{{\mathcalligra{c}}\,}_{{2},{\underline{\varvec{\mathcal{V}}}}}}\left({\mathcalligra{w}}\,\right)\right)\right]}{1-\Phi \left(\begin{array}{c}\left\{1-{\nabla }_{{{\mathcalligra{a}}\,}_{2}}^{-1}\left[1-{\text{exp}}\left(-{{\mathcalligra{a}}\,}_{2}{\mathcal{G}}_{{\mathcalligra{b}\,\,}\,\,_{2},{{\mathcalligra{c}}\,}_{{2},{\underline{\varvec{\mathcal{V}}}}}}\left({\mathcalligra{w}}\,\right)\right)\right]\right\}\\ \times \left\{1-{\nabla }_{{{\mathcalligra{a}}\,}_{1}}^{-1}\left[1-{\text{exp}}\left(-{{\mathcalligra{a}}\,}_{1}{\mathcal{G}}_{{\mathcalligra{b}\,\,}\,\,_{1},{{\mathcalligra{c}}\,}_{{1},{\underline{\varvec{\mathcal{V}}}}}}\left(\mathcalligra{m}\right)\right)\right]\right\}\end{array}\right)}|\Phi \in \left(-\mathrm{1,1}\right).$$

### The MvGPW-G type

The CTN type copula is recognized as one of the most user-friendly copulas for constructing multivariate distributions or families. Its ease of mathematical formulation and application make it an attractive choice. In practical applications, particularly in engineering, medical journals, insurance, reinsurance, and various other domains, multivariate distributions or families built upon the CTN copula offer flexibility and find versatile use in modeling multivariate data.

Following the principles of the CTN type copula, then the multivariate GPW-G type model can be derived as 

27 $$H({\mathbb{S}}_{\,\mathcalligra{u}\,\,})={\left(\sum \limits_{\,\mathcalligra{u}\,\,=1}^{\,\mathcalligra{v}\,}{\left\{{\nabla }_{{\mathcalligra{a}}\,_{\,\mathcalligra{u}\,\,}}^{-1}\left[1-{\text{exp}}\left(-{\mathcalligra{a}}\,_{\,\mathcalligra{u}\,\,}{\mathcal{G}}_{{{\mathcalligra{b}\,\,}\,\,_{\,\mathcalligra{u}\,\,}},{{\mathcalligra{c}}\,_{\,\mathcalligra{u}\,\,}},{\underline{\varvec{\mathcal{V}}}}}\left(\mathcalligra{m}\right)\right)\right]\right\}}^{-\Phi }+1-\,\mathcalligra{v}\,\right)}^{-\frac{1}{\Phi }}.$$

There is potential for future independent research endeavors to delve into several of these binary and multivariate distributions, exploring their applications in crucial domains such as reliability, engineering, healthcare, insurance, re-insurance and actuarial sciences, among others. This avenue of research is promising because the current study has certain limitations that restrict the scope of such investigations.

## Main mathematical properties

When exploring new families of continuous distributions, it is of paramount importance to conduct a comprehensive examination of all their mathematical and statistical properties. This initial step is crucial before parameter estimation and prior to applying these new families in mathematical and statistical modeling and real life applications. Through the investigation of mathematical properties, we gain valuable insights into the characteristics of the new family, facilitating its seamless integration into statistical and mathematical modeling processes. Symbolic computing software systems, such as Mathematica and Maple, have enhanced capabilities for handling complicated formulae that arise in this research as a result of their expertise in processing sophisticated expressions. These characteristics allow these software systems to manage complex formulas that arise as a result of this research. When compared to the indirect computing method, which makes use of explicitly derived expressions that have been developed and perfected over the course of some amount of time, the direct calculation of statistical measures through the use of numerical integration may demonstrate a lower level of efficiency than the indirect computation method. Below we will present several sports characteristics with appropriate mathematical and statistical analysis for each characteristic separately.

### Simple re-presentation

We shall discuss a significant theorem and provide a mathematical proof below. We will obtain numerous new, pertinent mathematical results by applying this approach.

#### Theorem 1

If $W$ is a continuous type RV having the presented PDF in (4), i.e., $W$ ∼GPW-G (${\underline{\varvec{\mathcal{P}}}}$), then

28 $${f}_{{\underline{\varvec{\mathcal{P}}}}}\left({\mathcalligra{w}}\,\right){\underline{\varvec{\mathcal{P}}}}={\left({\mathcalligra{a}}\,,\mathcalligra{b}\,\,,{\mathcalligra{c}}\,,{{\underline{\varvec{\mathcal{V}}}}}^{{\text{T}}}\right)}^{{\text{T}}}=\sum \limits_{{\,\mathcalligra{q}\,},{\,\mathcalligra{j}\,}=0}^{\infty }{D}_{{\,\mathcalligra{q}\,},{\,\mathcalligra{j}\,}}{{\varvec{\mathcalligra{h}\,}}}\,\,_{{{\mathcalligra{c}}\,}^{*}}\left({\mathcalligra{w}}\,\right){|}_{{{\mathcalligra{c}}\,}^{*}=\left[{\,\mathcalligra{q}\,}+1\right]{\mathcalligra{c}}\,+{\,\mathcalligra{j}\,}},$$

where ${{\varvec{\mathcalligra{h}\,}}}\,\,_{{{\mathcalligra{c}}\,}^{*}}\left({\mathcalligra{w}}\,\right)=d{\varvec{\mathcal{H}}}_{{{\mathcalligra{c}}\,}^{*}}\left({\mathcalligra{w}}\,\right)/d{\mathcalligra{w}}\,={{\mathcalligra{c}}\,}^{*}{\varvec{\mathcalligra{h}\,}}({\mathcalligra{w}}\,){\varvec{\mathcal{H}}}_{{\underline{\varvec{\mathcal{V}}}}}{\left({\mathcalligra{w}}\,\right)}^{{{\mathcalligra{c}}\,}^{*}-1}$ is the EXP-G PDF with power parameter ${{\mathcalligra{c}}\,}^{*}$ and

$${D}_{{\,\mathcalligra{q}\,},{\,\mathcalligra{j}\,}}=\sum \limits_{{\,\mathcalligra{v}\,},{\,\mathcalligra{u}\,\,}=0}^{+\infty }{{{\mathcalligra{a}}\,}^{1+{\,\mathcalligra{v}\,}}\mathcalligra{b}\,\,{\mathcalligra{c}}\,\nabla }_{{\mathcalligra{a}}\,}^{-1}\frac{{\left(-1\right)}^{{\,\mathcalligra{v}\,}+{\,\mathcalligra{q}\,}+{\,\mathcalligra{u}\,\,}}{\left({\,\mathcalligra{u}\,\,}+1\right)}^{{\,\mathcalligra{q}\,}}\Gamma \left(\mathcalligra{b}\,\,\left({\,\mathcalligra{v}\,}+1\right)\right)\Gamma \left({{\mathcalligra{c}}\,}^{*}+1\right)}{{\,\mathcalligra{v}\,}!{\,\mathcalligra{u}\,\,}!{\,\mathcalligra{q}\,}!{\,\mathcalligra{j}\,}!{{\mathcalligra{c}}\,}^{*}\Gamma \left(\mathcalligra{b}\,\,\left({\,\mathcalligra{v}\,}+1\right)-{\,\mathcalligra{u}\,\,}\right)\Gamma \left(\left[{\,\mathcalligra{q}\,}+1\right]{\mathcalligra{c}}\,+1\right)}.$$

#### Proof

First, by using the common power series function for $\exp\left(.\right)$, we expand the quantity ${A}_{{\,\mathcalligra{u}\,\,}}\left({\mathcalligra{w}}\,\right)$ where

$${A}_{{\,\mathcalligra{u}\,\,}}\left({\mathcalligra{w}}\,\right)={\text{exp}}\left(-{\mathcalligra{a}}\,{\left\{1-{\text{exp}}\left[-{\varvec{\mathcal{U}}}_{{\mathcalligra{c}}\,,{\underline{\varvec{\mathcal{V}}}}}\left({\mathcalligra{w}}\,\right)\right]\right\}}^{\mathcalligra{b}\,\,}\,\,\,\,\right).$$

Then, the PDF in (4) can then be expressed as

29 $${f}_{{\underline{\varvec{\mathcal{P}}}}}\left({\mathcalligra{w}}\,\right)|{\underline{\varvec{\mathcal{P}}}}={\left({\mathcalligra{a}}\,,\mathcalligra{b}\,\,,{\mathcalligra{c}}\,,{{\underline{\varvec{\mathcal{V}}}}}^{{\text{T}}}\right)}^{{\text{T}}}={\nabla }_{{\mathcalligra{a}}\,}^{-1}{\mathcalligra{c}}\,\mathcalligra{b}\,\,\sum \limits_{{\,\mathcalligra{v}\,}=0}^{+\infty }\frac{{\left(-1\right)}^{{\,\mathcalligra{v}\,}}{{\mathcalligra{a}}\,}^{1+{\,\mathcalligra{v}\,}}{\exp}\left[-{\varvec{\mathcal{U}}}_{{\mathcalligra{c}}\,,{\underline{\varvec{\mathcal{V}}}}}\left({\mathcalligra{w}}\,\right)\right]}{{\,\mathcalligra{v}\,}!{\overline{\varvec{\mathcal{H}}}}_{{\underline{\varvec{\mathcal{V}}}}}{\left({\mathcalligra{w}}\,\right)}^{{\mathcalligra{c}}\,+1}{\varvec{\mathcal{H}}}_{{\underline{\varvec{\mathcal{V}}}}}{\left({\mathcalligra{w}}\,\right)}^{-{\mathcalligra{c}}\,+1}}{{{\varvec{\mathcalligra{h}\,}}}\,\,_{{\underline{\varvec{\mathcal{V}}}}}\left({\mathcalligra{w}}\,\right)\left\{1-\exp\left[-{\varvec{\mathcal{U}}}_{{\mathcalligra{c}}\,,{\underline{\varvec{\mathcal{V}}}}}\left({\mathcalligra{w}}\,\right)\right]\right\}}^{\mathcalligra{b}\,\,\left({\,\mathcalligra{v}\,}+1\right)-1}.$$

Then, consider the power series

30 $${\left(1-\frac{{\gamma }_{1}}{{\gamma }_{2}}\right)}^{{\gamma }_{3}}=\sum \limits_{{\gamma }_{4}=0}^{+\infty }{\left(-1\right)}^{{\gamma }_{4}}\frac{\Gamma \left(1+{\gamma }_{3}\right)}{{\gamma }_{4}!\Gamma \left(1+{\gamma }_{3}-{\gamma }_{4}\right)}{\left(\frac{{\gamma }_{1}}{{\gamma }_{2}}\right)}^{{\gamma }_{4}}{|}_{\left|\frac{{\gamma }_{1}}{{\gamma }_{2}}\right|<1\text{ and }{\gamma }_{3}>0}.$$

Applying (30) to the quantity ${B}_{{\,\mathcalligra{u}\,\,}}\left({\mathcalligra{w}}\,\right)$ where

$${B}_{{\,\mathcalligra{u}\,\,}}\left({\mathcalligra{w}}\,\right)={\left\{1-{\text{exp}}\left[-{\varvec{\mathcal{U}}}_{{\mathcalligra{c}}\,,{\underline{\varvec{\mathcal{V}}}}}\left({\mathcalligra{w}}\,\right)\right]\right\}}^{\mathcalligra{b}\,\,\left({\,\mathcalligra{v}\,}+1\right)-1},$$

we get

31 $$\begin{aligned}{f}_{{\underline{\varvec{\mathcal{P}}}}}\left({\mathcalligra{w}}\,\right)|{\underline{\varvec{\mathcal{P}}}}&={\left({\mathcalligra{a}}\,,\mathcalligra{b}\,\,,{\mathcalligra{c}}\,,{{\underline{\varvec{\mathcal{V}}}}}^{{\text{T}}}\right)}^{{\text{T}}}={\nabla }_{{\mathcalligra{a}}\,}^{-1}{\mathcalligra{c}}\,\mathcalligra{b}\,\,{{\varvec{\mathcalligra{h}\,}}}\,\,_{{\underline{\varvec{\mathcal{V}}}}}\left({\mathcalligra{w}}\,\right)\frac{{\varvec{\mathcal{H}}}_{{\underline{\varvec{\mathcal{V}}}}}{\left({\mathcalligra{w}}\,\right)}^{{\mathcalligra{c}}\,-1}}{{\overline{\varvec{\mathcal{H}}}}_{{\underline{\varvec{\mathcal{V}}}}}{\left({\mathcalligra{w}}\,\right)}^{{\mathcalligra{c}}\,+1}}\sum \limits_{{\,\mathcalligra{v}\,},{\,\mathcalligra{u}\,\,}=0}^{+\infty }{{\mathcalligra{a}}\,}^{1+{\,\mathcalligra{v}\,}}\frac{{\left(-1\right)}^{{\,\mathcalligra{v}\,}+{\,\mathcalligra{u}\,\,}}\Gamma \left(\mathcalligra{b}\,\,\left({\,\mathcalligra{v}\,}+1\right)\right)}{{\,\mathcalligra{u}\,\,}!{\,\mathcalligra{v}\,}!\Gamma \left(\mathcalligra{b}\,\,\left({\,\mathcalligra{v}\,}+1\right)-{\,\mathcalligra{u}\,\,}\right)}\\ &\quad\times {\text{exp}}\left[-\left({\,\mathcalligra{u}\,\,}+1\right){\varvec{\mathcal{U}}}_{{\mathcalligra{c}}\,,{\underline{\varvec{\mathcal{V}}}}}\left({\mathcalligra{w}}\,\right)\right].\end{aligned}$$

Then, if

$${C}_{{\,\mathcalligra{u}\,\,}}\left({\mathcalligra{w}}\,\right)=\exp\left[-\left({\,\mathcalligra{u}\,\,}+1\right){\varvec{\mathcal{U}}}_{{\mathcalligra{c}}\,,{\underline{\varvec{\mathcal{V}}}}}\left({\mathcalligra{w}}\,\right)\right],$$

we have

32 $${C}_{{\,\mathcalligra{u}\,\,}}\left({\mathcalligra{w}}\,\right)=\sum \limits_{{\,\mathcalligra{q}\,}=0}^{+\infty }{\left(-1\right)}^{{\,\mathcalligra{q}\,}}\frac{1}{{\,\mathcalligra{q}\,}!}\frac{{\varvec{\mathcal{H}}}_{{\underline{\varvec{\mathcal{V}}}}}{\left({\mathcalligra{w}}\,\right)}^{{\,\mathcalligra{q}\,}{\mathcalligra{c}}\,}}{{\overline{\varvec{\mathcal{H}}}}_{{\underline{\varvec{\mathcal{V}}}}}{\left({\mathcalligra{w}}\,\right)}^{{\,\mathcalligra{q}\,}{\mathcalligra{c}}\,}}{\left({\,\mathcalligra{u}\,\,}+1\right)}^{{\,\mathcalligra{q}\,}}.$$

Inserting the (32) in (31), we have

33 $${f}_{{\underline{\varvec{\mathcal{P}}}}}\left({\mathcalligra{w}}\,\right)|{\underline{\varvec{\mathcal{P}}}}={\left({\mathcalligra{a}}\,,\mathcalligra{b}\,\,,{\mathcalligra{c}}\,,{{\underline{\varvec{\mathcal{V}}}}}^{{\text{T}}}\right)}^{{\text{T}}}=\mathcalligra{b}\,\,{\mathcalligra{c}}\,{\nabla }_{{\mathcalligra{a}}\,}^{-1}\hspace{0.33em}\sum \limits_{{\,\mathcalligra{v}\,},{\,\mathcalligra{u}\,\,},{\,\mathcalligra{q}\,}=0}^{+\infty }{{\mathcalligra{a}}\,}^{1+{\,\mathcalligra{v}\,}}{\left(-1\right)}^{{\,\mathcalligra{v}\,}+{\,\mathcalligra{q}\,}+{\,\mathcalligra{u}\,\,}}\frac{\Gamma \left(\mathcalligra{b}\,\,\left({\,\mathcalligra{v}\,}+1\right)\right){\left({\,\mathcalligra{u}\,\,}+1\right)}^{{\,\mathcalligra{q}\,}}}{{\,\mathcalligra{v}\,}!{\,\mathcalligra{u}\,\,}!{\,\mathcalligra{q}\,}!\Gamma \left(\mathcalligra{b}\,\,\left({\,\mathcalligra{v}\,}+1\right)-{\,\mathcalligra{u}\,\,}\right)}{{\varvec{\mathcalligra{h}\,}}}\,\,_{{\underline{\varvec{\mathcal{V}}}}}\left({\mathcalligra{w}}\,\right)\frac{{\varvec{\mathcal{H}}}_{{\underline{\varvec{\mathcal{V}}}}}{\left({\mathcalligra{w}}\,\right)}^{\left({\,\mathcalligra{q}\,}+1\right){\mathcalligra{c}}\,-1}}{{\overline{\varvec{\mathcal{H}}}}_{{\underline{\varvec{\mathcal{V}}}}}{\left({\mathcalligra{w}}\,\right)}^{\left({\,\mathcalligra{q}\,}+1\right){\mathcalligra{c}}\,+1}}.$$

Then,

34 $${\left[1-{\varvec{\mathcal{H}}}_{\underline{\xi }}\left({\mathcalligra{w}}\,\right)\right]}^{-\left[\left({\,\mathcalligra{q}\,}+1\right){\mathcalligra{c}}\,+1\right]}=\sum \limits_{{\,\mathcalligra{j}\,}=0}^{+\infty }\frac{1}{{\,\mathcalligra{j}\,}!\Gamma \left(\left[{\,\mathcalligra{q}\,}+1\right]{\mathcalligra{c}}\,+1\right)}\Gamma \left(\left[{\,\mathcalligra{q}\,}+1\right]{\mathcalligra{c}}\,+{\,\mathcalligra{j}\,}+1\right){\varvec{\mathcal{H}}}_{{\underline{\varvec{\mathcal{V}}}}}{\left({\mathcalligra{w}}\,\right)}^{{\,\mathcalligra{j}\,}}.$$

Inserting (34) in (33), we get

$${f}_{{\underline{\varvec{\mathcal{P}}}}}\left({\mathcalligra{w}}\,\right)|{\underline{\varvec{\mathcal{P}}}}={\left({\mathcalligra{a}}\,,\mathcalligra{b}\,\,,{\mathcalligra{c}}\,,{{\underline{\varvec{\mathcal{V}}}}}^{{\text{T}}}\right)}^{{\text{T}}}=\sum \limits_{{\,\mathcalligra{q}\,},{\,\mathcalligra{j}\,}=0}^{+\infty }{D}_{{\,\mathcalligra{q}\,},{\,\mathcalligra{j}\,}}{{\varvec{\mathcalligra{h}\,}}}\,\,_{{{\mathcalligra{c}}\,}^{*}}\left({\mathcalligra{w}}\,\right){|}_{{{\mathcalligra{c}}\,}^{*}=\left[{\,\mathcalligra{q}\,}+1\right]{\mathcalligra{c}}\,+{\,\mathcalligra{j}\,}},$$

where ${{\varvec{\mathcalligra{h}\,}}}\,\,_{{{\mathcalligra{c}}\,}^{*}}\left({\mathcalligra{w}}\,\right)=d{\varvec{\mathcal{H}}}_{{{\mathcalligra{c}}\,}^{*}}\left({\mathcalligra{w}}\,\right)/d{\mathcalligra{w}}\,={{\mathcalligra{c}}\,}^{*}{\varvec{\mathcalligra{h}\,}}({\mathcalligra{w}}\,){\varvec{\mathcal{H}}}_{{\underline{\varvec{\mathcal{V}}}}}{\left({\mathcalligra{w}}\,\right)}^{{{\mathcalligra{c}}\,}^{*}-1}$ is the EXP-G PDFs with power parameter ${{\mathcalligra{c}}\,}^{*}$ and

35 $${D}_{{\,\mathcalligra{q}\,},{\,\mathcalligra{j}\,}}=\sum \limits_{{\,\mathcalligra{v}\,},{\,\mathcalligra{u}\,\,}=0}^{+\infty }{{{\mathcalligra{a}}\,}^{1+{\,\mathcalligra{v}\,}}\mathcalligra{b}\,\,{\mathcalligra{c}}\,\nabla }_{{\mathcalligra{a}}\,}^{-1}{\left(-1\right)}^{{\,\mathcalligra{v}\,}+{\,\mathcalligra{q}\,}+{\,\mathcalligra{u}\,\,}}{\left({\,\mathcalligra{u}\,\,}+1\right)}^{{\,\mathcalligra{q}\,}}\frac{\Gamma \left(\mathcalligra{b}\,\,\left({\,\mathcalligra{v}\,}+1\right)\right)\Gamma \left({{\mathcalligra{c}}\,}^{*}+1\right)}{{\,\mathcalligra{v}\,}!{\,\mathcalligra{u}\,\,}!{\,\mathcalligra{q}\,}!{\,\mathcalligra{j}\,}!{{\mathcalligra{c}}\,}^{*}\Gamma \left(\mathcalligra{b}\,\,\left({\,\mathcalligra{v}\,}+1\right)-{\,\mathcalligra{u}\,\,}\right)\Gamma \left(\left[{\,\mathcalligra{q}\,}+1\right]{\mathcalligra{c}}\,+1\right)}.$$

Because of Eq. (28), the probability density function (PDF) associated to the GPW-G class can be expressed as a linear formula for the PDFs that are associated with the exponentiated G (EXP-G) family. This is made possible by the fact that the two families are related. For this reason, it is absolutely necessary to have a complete comprehension of the characteristics/properties of the EXP-G distributions in order to conduct an exhaustive investigation into the many mathematical components of the new class. It is important to note that the CDFs of both GPW-G and EXP-G can also be represented as linear combinations of CDFs by making use of the formula that has been supplied. This is something that should be brought to your attention. Understanding the EXP-G distribution in its entirety is a prerequisite for the investigation and comprehension of the GPW-G family as well as the mathematical properties that are associated with it.

36 $${F}_{{\underline{\varvec{\mathcal{P}}}}}\left({\mathcalligra{w}}\,\right)|{\underline{\varvec{\mathcal{P}}}}={\left({\mathcalligra{a}}\,,\mathcalligra{b}\,\,,{\mathcalligra{c}}\,,{{\underline{\varvec{\mathcal{V}}}}}^{{\text{T}}}\right)}^{{\text{T}}}=\sum \limits_{{\,\mathcalligra{q}\,},{\,\mathcalligra{j}\,}=0}^{+\infty }{D}_{{\,\mathcalligra{q}\,},{\,\mathcalligra{j}\,}}\hspace{0.33em}{\varvec{\mathcal{H}}}_{{{\mathcalligra{c}}\,}^{*}}\left({\mathcalligra{w}}\,\right),$$

The ceriated function ${\varvec{\mathcal{H}}}_{{{\mathcalligra{c}}\,}^{*}}\left({\mathcalligra{w}}\,\right)$ is the CDF of the EXP-G but with power parameter ${{\mathcalligra{c}}\,}^{*}$.

### Moments

Following results of the Theorem 1, the $r$ th moment of $W$, say ${u}_{r,W}^{\prime}$, follows from Eq. (35) as

37 $${u}_{r,W}^{\prime}=E({W}^{r})=\sum \limits_{{\,\mathcalligra{q}\,},{\,\mathcalligra{j}\,}=0}^{+\infty }{D}_{{\,\mathcalligra{q}\,},{\,\mathcalligra{j}\,}}E({\mathcalligra{y}}_{{{\mathcalligra{c}}\,}^{*}}^{r}),$$

where ${\mathcalligra{y}}_{{{\mathcalligra{c}}\,}^{*}}$ denotes the EXP-G RV with the power parameter ${{\mathcalligra{c}}\,}^{*}$. The $\Delta$th central moment of $W$, say ${M}_{\Delta }$, is given by

38 $${M}_{\Delta ,W}=E{\left(W-{u}_{1}^{\prime}\right)}^{\Delta }=\sum \limits_{r=0}^{+\infty }\left(\begin{array}{c}\Delta \\ r\end{array}\right){\left(-{u}_{1}^{\prime}\right)}^{\Delta -r}E\left({W}^{r}\right)=\sum \limits_{r=0}^{+\infty }\sum \limits_{{\,\mathcalligra{q}\,},{\,\mathcalligra{j}\,}=0}^{+\infty }{\left(-{u}_{1}^{\prime}\right)}^{\Delta -r}{D}_{{\,\mathcalligra{q}\,},{\,\mathcalligra{j}\,}}\left(\begin{array}{c}\Delta \\ r\end{array}\right)E\left({\mathcalligra{y}}_{{{\mathcalligra{c}}\,}^{*}}^{r}\right).$$

### Moment generating function (MGF)

 It is clear that the most important strategy for obtaining the MGF can be deduced from Eq. (28), and it is presented in the following format:

39 $${M}_{W}(\mathcalligra{m})=\sum \limits_{{\,\mathcalligra{q}\,},{\,\mathcalligra{j}\,}=0}^{+\infty }{D}_{{\,\mathcalligra{q}\,},{\,\mathcalligra{j}\,}}{M}_{{{\mathcalligra{c}}\,}^{*}}\left(\mathcalligra{m}\right),$$

where the function ${M}_{{{\mathcalligra{c}}\,}^{*}}(\mathcalligra{m})$ is the simplest MGF of ${\mathcalligra{y}}_{{{\mathcalligra{c}}\,}^{*}}$ which can be easily derived and help us to get the MGF of the new family.

### Incomplete moments

Moments are useful in the field of physical science for a variety of applications, including the description of the distribution of objects in time and space, which is a component of the analysis of physical phenomena. On the other hand, incomplete moments are utilized in a variety of situations, one of which is image processing. Here, they explain the distribution of pixel intensities in images, which makes image analysis and enhancement much simpler. The ${\mathcalligra{s}}^{th}$ incomplete moment, say ${I}_{\mathcalligra{s},W}(\mathcalligra{m})$, of $W$ can be expressed from (28) as

40 $${I}_{\mathcalligra{s},W}(\mathcalligra{m})={\int }_{-\infty }^{\mathcalligra{m}}{{\mathcalligra{w}}\,}^{\mathcalligra{s}}f\left({\mathcalligra{w}}\,\right)d{\mathcalligra{w}}\,=\sum \limits_{{\,\mathcalligra{q}\,},{\,\mathcalligra{j}\,}=0}^{+\infty }{D}_{{\,\mathcalligra{q}\,},{\,\mathcalligra{j}\,}}{\int }_{-\infty }^{\mathcalligra{m}}{{\mathcalligra{w}}\,}^{\mathcalligra{s}}{{\varvec{\mathcalligra{h}\,}}}\,\,_{{{\mathcalligra{c}}\,}^{*}}\left({\mathcalligra{w}}\,\right)d{\mathcalligra{w}}\,.$$

Without a doubt, the integral that is depicted in Eq. (40) is capable of being calculated theoretically and numerically for the vast majority of the fundamental models. In more recent times, the availability of numerical approaches and easily accessible statistical software has substantially lessened the difficulties and inconveniencies that are connected with a wide variety of statistical functions. Therefore, numerical analyses have developed into an essential part of the modern research being conducted in the field of probability distribution theory.

## Estimation method

Classical estimation techniques, such as maximal likelihood estimation (MLE) and method of moments estimation, are fundamental in the field of statistics and have been subject to thorough examination. Nonetheless, the efficacy of these methods can be impacted by a range of factors, including the size of the sample, the assumed distribution of the data, and the existence of outliers or influential data points. Simulation studies provide a valuable approach to evaluate and compare the efficacy of conventional estimating methods across various circumstances. Various investigations facilitate researchers in acquiring insights into the behavior of various techniques under varying settings and enable a comprehensive assessment of their resilience, particularly when their fundamental assumptions are violated. For example, in situations when the data exhibits non-normal distribution, the utilization of maximum likelihood estimation may not be the most appropriate option. In such situations, it may be advantageous to consider alternatives such as robust estimating or nonparametric approaches, as they have the potential to offer more robustness and accuracy. Simulation studies offer a methodical strategy for assessing the efficacy of these alternative methodologies across diverse scenarios, thereby enabling comparisons with conventional procedures. To summarize, simulation studies are regarded as a valuable instrument for researchers, enabling them to carefully investigate and choose the most suitable estimating method, taking into account the distinct characteristics and complexities presented by the data under analysis. This methodology improves the dependability and precision of statistical analysis across various contexts.

Let ${{\mathcalligra{w}}\,}_{1},{{\mathcalligra{w}}\,}_{2},\dots ,{{\mathcalligra{w}}\,}_{\Delta }$ be certain sample randomly drown from our GPW-G family with its corresponding parameters ${\mathcalligra{a}}\,,\mathcalligra{b}\,\,,{\mathcalligra{c}}\,$, ${{\underline{\varvec{\mathcal{V}}}}}^{{\text{T}}}$ and let ${\underline{\varvec{\mathcal{P}}}}={\left({\mathcalligra{a}}\,,\mathcalligra{b}\,\,,{\mathcalligra{c}}\,,{{\underline{\varvec{\mathcal{V}}}}}^{{\text{T}}}\right)}^{{\text{T}}}$ be the corresponding parameter vector. Then, the log-likelihood function ${\mathcalligra{l}}_{{\underline{\varvec{\mathcal{P}}}}}$ for the GPW-G distribution can expressed as:

41 $$\begin{aligned}{{\mathcalligra{l}}_{\underline{\varvec{\mathcal{P}}}}}|{\underline{\varvec{\mathcal{P}}}}&={\left({\mathcalligra{a}}\,,\mathcalligra{b}\,\,,{\mathcalligra{c}}\,,{{\underline{\varvec{\mathcal{V}}}}}^{{\text{T}}}\right)}^{{\text{T}}}=\Delta {\text{log}}{\mathcalligra{a}}\,+\Delta {\text{log}}{\mathcalligra{c}}\,+\Delta {\text{log}}\mathcalligra{b}\,\,-\Delta {\text{log}}{\nabla }_{{\mathcalligra{a}}\,}+\sum \limits_{{\,\mathcalligra{u}\,\,}=1}^{\Delta }{\text{log}}{{\varvec{\mathcalligra{h}\,}}}\,\,_{{\underline{\varvec{\mathcal{V}}}}}\left({{\mathcalligra{w}}\,}_{{\,\mathcalligra{u}\,\,}}\right)\\ &\quad+\left({\mathcalligra{c}}\,-1\right)\sum \limits_{{\,\mathcalligra{u}\,\,}=1}^{\Delta }{\text{log}}{\varvec{\mathcal{H}}}_{{\underline{\varvec{\mathcal{V}}}}}\left({{\mathcalligra{w}}\,}_{{\,\mathcalligra{u}\,\,}}\right)-\left(1+{\mathcalligra{c}}\,\right)\sum \limits_{{\,\mathcalligra{u}\,\,}=1}^{\Delta }{\overline{\varvec{\mathcal{H}}}}_{{\underline{\varvec{\mathcal{V}}}}}\left({{\mathcalligra{w}}\,}_{{\,\mathcalligra{u}\,\,}}\right)-\sum \limits_{{\,\mathcalligra{u}\,\,}=1}^{\Delta }{\varvec{\mathcal{U}}}_{{\mathcalligra{c}}\,,{\underline{\varvec{\mathcal{V}}}}}\left({{\mathcalligra{w}}\,}_{{\,\mathcalligra{u}\,\,}}\right)\\ &\quad-\left(1-\mathcalligra{b}\,\,\right)\sum \limits_{{\,\mathcalligra{u}\,\,}=1}^{\Delta }\left\{1-{\text{exp}}\left[-{\varvec{\mathcal{U}}}_{{\mathcalligra{c}}\,,{\underline{\varvec{\mathcal{V}}}}}\left({{\mathcalligra{w}}\,}_{{\,\mathcalligra{u}\,\,}}\right)\right]\right\}-{\mathcalligra{a}}\,\sum \limits_{{\,\mathcalligra{u}\,\,}=1}^{\Delta }{\left\{1-{\text{exp}}\left[-{\varvec{\mathcal{U}}}_{{\mathcalligra{c}}\,,{\underline{\varvec{\mathcal{V}}}}}\left({{\mathcalligra{w}}\,}_{{\,\mathcalligra{u}\,\,}}\right)\right]\right\}}^{\mathcalligra{b}\,\,}\,\,\,\,.\end{aligned}$$

The maximum of this function can be accomplished by making use of appropriate software or by finding a solution to the set of nonlinear equations that are obtained from the differentiation of ${{\mathcalligra{l}}_{\underline{\varvec{\mathcal{P}}}}}$. Both of these methods are viable options. The following is a complete list of the components that make up the score vector in every situation:

42 $${{\textbf{U}}_{\mathcalligra{a}}\,}\left({{\mathcalligra{l}}_{\underline{\varvec{\mathcal{P}}}}}\right)=\frac{\partial }{\partial {\mathcalligra{a}}\,}{{\mathcalligra{l}}_{\underline{\varvec{\mathcal{P}}}}}=\frac{1}{{\mathcalligra{a}}\,} \Delta - \Delta \frac{ {\text{exp}}(-{\mathcalligra{a}}\,)}{{\nabla }_{\mathcalligra{a}}\,} -\sum \limits_{{{\,\mathcalligra{u}\,\,}=1}^{\Delta }}{\left\{1-{\text{exp}}\left[-{{\varvec{\mathcal{U}}}_{{\mathcalligra{c}}\,,{\underline{\varvec{\xi}}}}}\left({{\mathcalligra{w}}\,_{\,\mathcalligra{u}\,\,}}\right)\right]\right\}}^{\mathcalligra{b}\,\,}\,\,\,\,,$$

43 $$\begin{aligned}{\textbf{U}}_{\mathcalligra{b}}\left({\mathcalligra{l}}_{\underline{\varvec{\mathcal{P}}}}\right)&=\frac{\partial }{{\partial \mathcalligra{b}}}{\mathcalligra{l}}_{\underline{\varvec{\mathcal{P}}}}=\frac{1}{{\mathcalligra{b}}} \Delta +\sum \limits_{{\mathcalligra{u}\,\,}=1}^{\Delta }\left\{1-\exp\left[-{\varvec{\mathcal{U}}}_{{\mathcalligra{c}}\,,{\underline{\varvec{\xi}}}}\left({{\mathcalligra{w}}\,}_{{\,\mathcalligra{u}\,\,}}\right)\right]\right\}\\ &\quad-{\mathcalligra{a}}\,\sum \limits_{{\,\mathcalligra{u}\,\,}=1}^{\Delta }{\left\{1-\exp\left[-{{\varvec{O}}}_{{\mathcalligra{c}}\,,{\underline{\varvec{\xi}}}}\left({{\mathcalligra{w}}\,}_{{\,\mathcalligra{u}\,\,}}\right)\right]\right\}}^{\mathcalligra{b}\,\,}\,\,\,\,\log\left\{1-\exp\left[-{\mathcal{U}}_{{\mathcalligra{c}}\,,{\underline{\varvec{\mathcal{V}}}}}\left({{\mathcalligra{w}}\,}_{{\,\mathcalligra{u}\,\,}}\right)\right]\right\},\end{aligned}$$

44 $$\begin{aligned}{\textbf{U}}_{{\mathcalligra{c}}\,}\left({\mathcalligra{l}}_{\underline{\varvec{\mathcal{P}}}}\right)&=\frac{\partial }{\partial {\mathcalligra{c}}\,}{\mathcalligra{l}}_{\underline{\varvec{\mathcal{P}}}}=\frac{1}{{\mathcalligra{c}}\,}\Delta +{\sum }_{{\,\mathcalligra{u}\,\,}=1}^{\Delta }{\log}{\varvec{\Pi }}_{\underline{{\varvec{\xi}}}}\left({{\mathcalligra{w}}\,}_{{\,\mathcalligra{u}\,\,}}\right)-{\sum }_{{\,\mathcalligra{u}\,\,}=1}^{\Delta }{\varvec{\mathcal{U}}}_{{\mathcalligra{c}}\,,{\underline{\varvec{\mathcal{V}}}}}\left({\mathcalligra{w}}\,\right)\log{\varvec{\mathcal{U}}}_{1,{\underline{\varvec{\mathcal{V}}}}}\left({\mathcalligra{w}}\,\right)-{\sum }_{{\,\mathcalligra{u}\,\,}=1}^{\Delta }{\overline{\varvec{\Pi }}}_{\underline{{\varvec{\xi}}}}\left({{\mathcalligra{w}}\,}_{{\,\mathcalligra{u}\,\,}}\right)\\ &\quad-\left(1-\mathcalligra{b}\,\,\right){\sum }_{{\,\mathcalligra{u}\,\,}=1}^{\Delta }\exp\left[-{\varvec{\mathcal{U}}}_{{\mathcalligra{c}}\,,{\underline{\varvec{\xi}}}}\left({{\mathcalligra{w}}\,}_{{\,\mathcalligra{u}\,\,}}\right)\right]{\varvec{\mathcal{U}}}_{{\mathcalligra{c}}\,,\underline{\mathcal{V}}}\left({\mathcalligra{w}}\,\right)\log{\varvec{\mathcal{U}}}_{1,\underline{\mathcal{V}}}\left({\mathcalligra{w}}\,\right)\\ &\quad-{\mathcalligra{a}}\,\mathcalligra{b}\,\,{\sum }_{{\,\mathcalligra{u}\,\,}=1}^{\Delta }{\left\{1-\exp\left[-{\varvec{\mathcal{U}}}_{{\mathcalligra{c}}\,,{\underline{\varvec{\xi}}}}\left({{\mathcalligra{w}}\,}_{{\,\mathcalligra{u}\,\,}}\right)\right]\right\}}^{\mathcalligra{b}\,\,-1}\exp\left[-{{\varvec{O}}}_{{\mathcalligra{c}}\,,{\underline{\varvec{\xi}}}}\left({\mathcalligra{w}}\,_{\,\mathcalligra{u}\,\,}\right)\right]{\varvec{\mathcal{U}}}_{{\mathcalligra{c}}\,,{\underline{\varvec{\mathcal{V}}}}}\left({\mathcalligra{w}}\,\right)\log{\varvec{\mathcal{U}}}_{1,{\underline{\mathcal{V}}}}\left({\mathcalligra{w}}\,\right),\end{aligned}$$

and

45 $$\begin{aligned}{\textbf{U}}_{{\underline{\varvec{\mathcal{V}}}}_{\,\mathcalligra{q}\,}}\left({\mathcalligra{l}}_{\underline{\mathcal{P}}}\right)&=\frac{\partial }{\partial {\underline{\varvec{\mathcal{V}}}}_{\,\mathcalligra{q}\,}}{\mathcalligra{l}}_{\underline{\varvec{\mathcal{P}}}}=\sum \limits_{{\,\mathcalligra{u}\,\,}=1}^{\Delta }\frac{1}{{{\varvec{\mathcalligra{h}\,}}}\,\,_{\underline{{\varvec{\xi}}}}\left({{\mathcalligra{w}}\,}_{{\,\mathcalligra{u}\,\,}}\right)}{{\varvec{\mathcalligra{h}\,}}}\,\,_{\underline{{\varvec{\xi}}}}^{/}\left({{\mathcalligra{w}}\,}_{{\,\mathcalligra{u}\,\,}}\right)+\left({\mathcalligra{c}}\,-1\right)\sum \limits_{{\,\mathcalligra{u}\,\,}=1}^{\Delta }\frac{1}{{\varvec{\mathcal{H}}}_{\underline{{\varvec{\xi}}}}\left({{\mathcalligra{w}}\,}_{{\,\mathcalligra{u}\,\,}}\right)}{\varvec{\mathcal{H}}}_{\underline{{\varvec{\xi}}}}^{/}\left({{\mathcalligra{w}}\,}_{{\,\mathcalligra{u}\,\,}}\right)\\ &\quad\pm {\mathcalligra{c}}\,\sum 
\limits_{{\,\mathcalligra{u}\,\,}=1}^{\Delta }{\varvec{\mathcal{U}}}_{{\mathcalligra{c}}\,-1,{\underline{\varvec{\mathcal{V}}}}}\left({\mathcalligra{w}}\,\right){\mathcalligra{s}}_{{\,\mathcalligra{u}\,\,}}+\left({\mathcalligra{c}}\,+1\right)\sum \limits_{{\,\mathcalligra{u}\,\,}=1}^{\Delta }{\varvec{\mathcal{H}}}_{\underline{\varvec{\xi}}}^{/}\left({{\mathcalligra{w}}\,}_{{\,\mathcalligra{u}\,\,}}\right)\\ &\quad-\left(1-\mathcalligra{b}\,\,\right){\mathcalligra{c}}\,\sum \limits_{{\,\mathcalligra{u}\,\,}=1}^{\Delta }{\text{exp}}\left[-{\varvec{\mathcal{U}}}_{{\mathcalligra{c}}\,,{\underline{\varvec{\xi}}}}\left({{\mathcalligra{w}}\,}_{{\,\mathcalligra{u}\,\,}}\right)\right]{\varvec{\mathcal{U}}}_{{\mathcalligra{c}}\,-1,{\underline{\varvec{\mathcal{V}}}}}\left({\mathcalligra{w}}\,\right){\mathcalligra{s}}_{{\,\mathcalligra{u}\,\,}}\\&\quad-{\mathcalligra{a}}\,\mathcalligra{b}\,\,{\mathcalligra{c}}\,\sum \limits_{{\,\mathcalligra{u}\,\,}=1}^{\Delta }{\left\{1-{\text{exp}}\left[-{\varvec{\mathcal{U}}}_{{\mathcalligra{c}}\,,\underline{{\varvec{\xi}}}}\left({{\mathcalligra{w}}\,}_{{\,\mathcalligra{u}\,\,}}\right)\right]\right\}}^{\mathcalligra{b}\,\,-1}{\text{exp}}\left[-{\varvec{\mathcal{U}}}_{{\mathcalligra{c}}\,,{\underline{\varvec{\xi}}}}\left({{\mathcalligra{w}}\,}_{{\,\mathcalligra{u}\,\,}}\right)\right]{\varvec{\mathcal{U}}}_{{\mathcalligra{c}}\,-1,{\underline{\varvec{\mathcal{V}}}}}\left({\mathcalligra{w}}\,\right){\mathcalligra{s}}_{{\,\mathcalligra{u}\,\,}},\end{aligned}$$

where

$${\mathcalligra{s}}_{{\,\mathcalligra{u}\,\,}}={{\varvec{\mathcal{H}}}_{\underline{\varvec{\xi}}}^{/}}\left({{\mathcalligra{w}}\,}_{{\,\mathcalligra{u}\,\,}}\right){\left({{\overline{\varvec{\mathcal{H}}}}_{\underline{\varvec{\xi}}}}\left({{\mathcalligra{w}}\,}_{{\,\mathcalligra{u}\,\,}}\right)\right)}^{-2},{{\varvec{\mathcalligra{h}\,}}_{\underline{\varvec{\xi}}}^{/}}\left({{\mathcalligra{w}}\,}_{{\,\mathcalligra{u}\,\,}}\right)=\frac{d}{d{{\underline{\varvec{\mathcal{V}}}}}_{{\,\mathcalligra{q}\,}}}{{\varvec{\mathcalligra{h}\,}}_{\underline{\varvec{\mathcal{V}}}}}\left({\mathcalligra{w}}\,\right)\, and\, {{\varvec{\mathcal{H}}}_{\underline{\varvec{\xi}}}^{/}}\left({{\mathcalligra{w}}\,}_{{\,\mathcalligra{u}\,\,}}\right)=\frac{d}{d{{\underline{\varvec{\mathcal{V}}}}}_{{\,\mathcalligra{q}\,}}}{{\varvec{\mathcal{H}}}_{\underline{\varvec{\mathcal{V}}}}}\left({\mathcalligra{w}}\,\right).$$

In this section, a comprehensive analysis is conducted on the outcomes and interpretations of these actual applications. The subsequent dialogues explore the knowledge obtained from these analyses, elucidating the practical consequences and significance of utilizing compound distributions with copulas in several fields. The aforementioned case studies provide tangible illustrations of the practical application of statistical methods in tackling real life difficulties and producing significant insights to inform decision-making and problem-solving endeavors.

## Special case

Consider the one parameter Lomax (LO) probability model as our considered base-line model for modeling and analyzing real-life datasets in the applications part and in “Simulation study” section, where

46 $${{\varvec{\mathcal{U}}}_{{\mathcalligra{c}}\,,\gamma}}\left({{\mathcalligra{w}}\,}\right)={\left[{\left(1+{{\mathcalligra{w}}\,}\right)}^{\gamma }-1\right]}^{{\mathcalligra{c}}\,}.$$

Then, the CDF of the GPWLO model can then be stated as, based on (3), which is as follows:

47 $${F}_{{\underline{\varvec{\mathcal{P}}}}}\left({\mathcalligra{w}}\,\right)|{\underline{\varvec{\mathcal{P}}}}={\left({\mathcalligra{a}}\,,\mathcalligra{b}\,\,,{\mathcalligra{c}}\,,\gamma \right)}^{{\text{T}}}={\nabla }_{{\mathcalligra{a}}\,}^{-1}\left[1-{\text{exp}}\left(-{\mathcalligra{a}}\,{\left\{1-{\text{exp}}\left[-{{\varvec{\mathcal{U}}}_{{\mathcalligra{c}}\,,\gamma }}\left({\mathcalligra{w}}\,\right)\right]\right\}}^{\mathcalligra{b}\,\,}\,\,\,\,\right)\right]{|}_{{\mathcalligra{w}}\,>0}.$$

**Figure 1:**
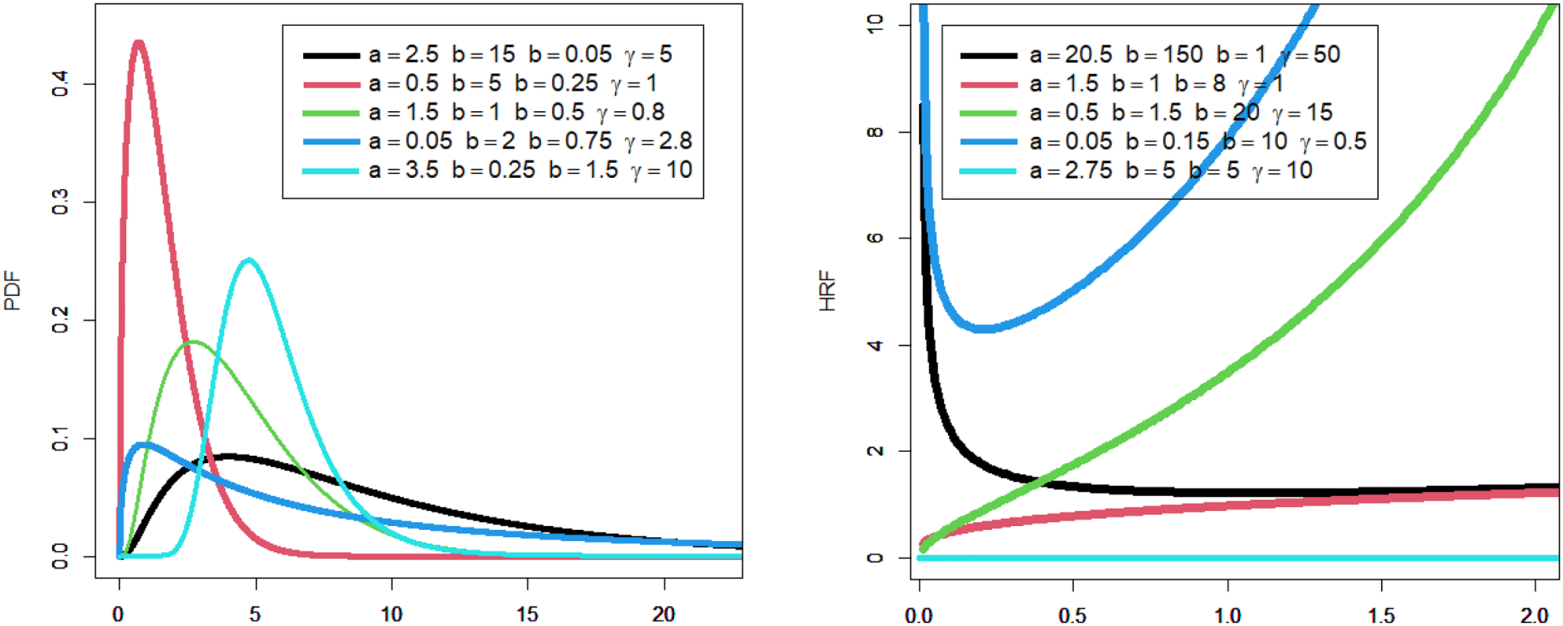
Some plots for the density of the new model due to some selected parameters values (left panel), some plots for the density of the new model due to some selected parameters values (right panel).

The corresponding PDF due to (47) can be easily derived be the differentiation of (47) or by using (4) directly.

48 $${f}_{{\underline{\varvec{\mathcal{P}}}}}\left({\mathcalligra{w}}\,\right)|{\underline{\varvec{\mathcal{P}}}}={\left({\mathcalligra{a}}\,,\mathcalligra{b}\,\,,{\mathcalligra{c}}\,,\gamma \right)}^{{\text{T}}}={\mathcalligra{a}}\,{\mathcalligra{c}}\,\mathcalligra{b}\,\,{\nabla }_{{\mathcalligra{a}}\,}^{-1}{\text{exp}}\left(-{\mathcalligra{a}}\,{\left\{1-{\text{exp}}\left[-{\varvec{\mathcal{U}}}_{{\mathcalligra{c}}\,,\gamma }\left({\mathcalligra{w}}\,\right)\right]\right\}}^{\mathcalligra{b}\,\,}\,\,\,\,\right)\times \frac{{\varvec{\mathcalligra{h}\,}}_{\gamma }\left({\mathcalligra{w}}\,\right){\varvec{\mathcal{H}}}_{\gamma }{\left({\mathcalligra{w}}\,\right)}^{{\mathcalligra{c}}\,-1}{\text{exp}}\left[-{\varvec{\mathcal{U}}}_{{\mathcalligra{c}}\,,\gamma }\left({\mathcalligra{w}}\,\right)\right]}{{\overline{\varvec{\mathcal{H}}}}_{\gamma }{\left({\mathcalligra{w}}\,\right)}^{{\mathcalligra{c}}\,+1}{\left\{1-{\text{exp}}\left[-{\varvec{\mathcal{U}}}_{{\mathcalligra{c}}\,,\gamma }\left({\mathcalligra{w}}\,\right)\right]\right\}}^{1-\mathcalligra{b}\,\,}},$$

where ${\varvec{\mathcal{U}}}_{{\mathcalligra{c}}\,,\gamma }\left({{\mathcalligra{w}}\,}\right)$ is defined in (46), ${\varvec{\mathcalligra{h}\,}}_{\gamma }\left({{\mathcalligra{w}}\,}\right)$ refers to the PDF of the LO base-line model, ${\varvec{\mathcal{H}}}_{\gamma }\left({\mathcalligra{w}}\,\right)$ refers to the CDF type of the LO base-line and ${\overline{\varvec{\mathcal{H}}}}_{\gamma }\left({\mathcalligra{w}}\,\right)$ is the survival function of the LO base-line model. The examination of the probability density function (PDF) is one among several statistical endeavors that can be executed by diverse methodologies, such as numerical and graphical approaches. This subsequent section will mostly center on the graphical representation as we examine the adaptability and importance of the newly developed density function. In addition, a graphical analysis of the failure/hazard rate function (HRF) will be conducted, as it holds significant importance in the context of the study. Furthermore, we will offer more profound observations and elucidate the recent discoveries. Figure 1 (left panel) displays density graphs for the new model, illustrating the impact of specific parameter values. Figure 1 (right panel) displays various plots illustrating the hemodynamic response function (HRF) of the novel model, corresponding to specific parameter values that have been chosen.

The observation that the new PDF can exhibit unimodality, characterized by a single peak that can assume diverse shapes, is readily apparent. Irrespective of the prevailing conditions, the fundamental form of the function consistently exhibits a prolonged rightward tail. This implies that the serious function is a strong candidate for utilization in situations involving data with a pronounced tail, as depicted in the left panel of Fig. 1. According to the information presented in the right panel of Fig. 1, the probability model’s hemodynamic response function (HRF) exhibits a flexible nature and encompasses several significant and practical shapes. These shapes include the decreasing constant HRF (as indicated in the first line), the increasing HRF (as observed in the second and third lines), the increasing-constant-decreasing HRF or U-HRF (as depicted in the fourth lines), and the constant HRF (as represented in the fifth line). The failure rate function is frequently linked to reliability theory and survival analysis within the realm of probability theory and statistics. This statement elucidates the manner in which a stochastic variable, typically denoting the temporal duration until a particular occurrence (such as the malfunction of a machine), experiences failure at various temporal intervals. When evaluating the resilience or dependability of a process or system, it may be essential to look at the shape of the failure rate function. A probability distribution’s resilience and adaptability in statistical modeling techniques and real-world applications across several domains are demonstrated by its capacity to tolerate various failure rate patterns.

## Simulation study

Simulation study plays an important role when studying and analyzing probability distributions for many reasons. Simulation allows validation of the probabilistic models used. When we infer a probabilistic model to real data, it can be difficult to determine its exact fit. Simulation gives you the opportunity to create artificial data using your probabilistic model and compare it to real data. Simulations are used to test certain hypotheses about a probabilistic model. One can load the model by making certain assumptions and then run a simulation to validate them. For example, you can test whether the probability distribution fits the actual data. In some cases, simulation can be used to estimate the parameters of a probabilistic model. For example, if you have noisy or insufficient data to estimate model parameters using traditional methods, you can use simulation to better estimate these parameters. Simulation can be used to estimate probabilities or conditional probabilities on events. This can be useful in risk analysis and decision making in many fields such as engineering, computer science, and finance.

In this paper, we can easily present a simulation study (numerical or graphical). Simulations allow the study of complex interactions and dynamic behavior of the new probabilistic family. They can be used to understand how probability distributions change over time or the effect of the interaction between several variables. Graphical simulations are used to assess the robustness of MLE estimators. By introducing different types of noise or data distributions, you can observe how the bias and MSE of MLE estimators change under various conditions. This helps in understanding how well MLE performs when data deviates from ideal assumptions. Using mean square errors (MSE) and biases as criteria for evaluating the behavior of estimators in the context of the MLE through graphical simulation is essential for various reasons:

I. MSE and bias provide measures of the accuracy of estimators. The bias quantifies how consistently an estimator overestimates or underestimates the true parameter value, while the MSE combines both bias and variance to give a comprehensive measure of overall accuracy. Graphical simulation allows you to visualize how these measures change as you adjust parameters or sample sizes, aiding in the assessment of estimator accuracy.
II. MLE estimators are known for achieving the Cramer–Rao lower bound (CRLB) efficiency under certain conditions. Graphical simulations can help you compare the efficiency of MLE estimators to other estimators by plotting their MSEs side by side. This helps identify which estimator is more efficient and thus has smaller MSE.
III. MLE estimators are consistent, meaning that they converge to the true parameter value as the sample size increases. By plotting MSE and bias as functions of sample size in graphical simulations, you can demonstrate and confirm the consistency property of MLE estimators.
IV. MSE and bias can be plotted against sample size to illustrate how the precision and bias of MLE estimators change as more data points are collected. This information is crucial for determining the required sample size for a specific estimation problem.
V. In cases where we are considering multiple models or parameterizations, graphical simulations can aid in model selection. By comparing the MSE and bias of MLE estimators under different models, you can identify which model provides the best balance between accuracy and bias.
VI. Graphical simulations are valuable tools for teaching and communicating statistical concepts. They provide intuitive visualizations that make it easier for students or colleagues to grasp the behavior of MLE estimators under different conditions.

For assessing the estimation method, we considered the following algorithm:

1. For $n=\mathrm{200,220},\dots ,500|\mathrm{N }=1000$, we generate samples from the GPWLO model.
2. For each sample, we compute the MLE.
3. For each sample, we compute standard errors (via inverting the information matrix) and then the biases of the MLEs.

**Figure 2:**
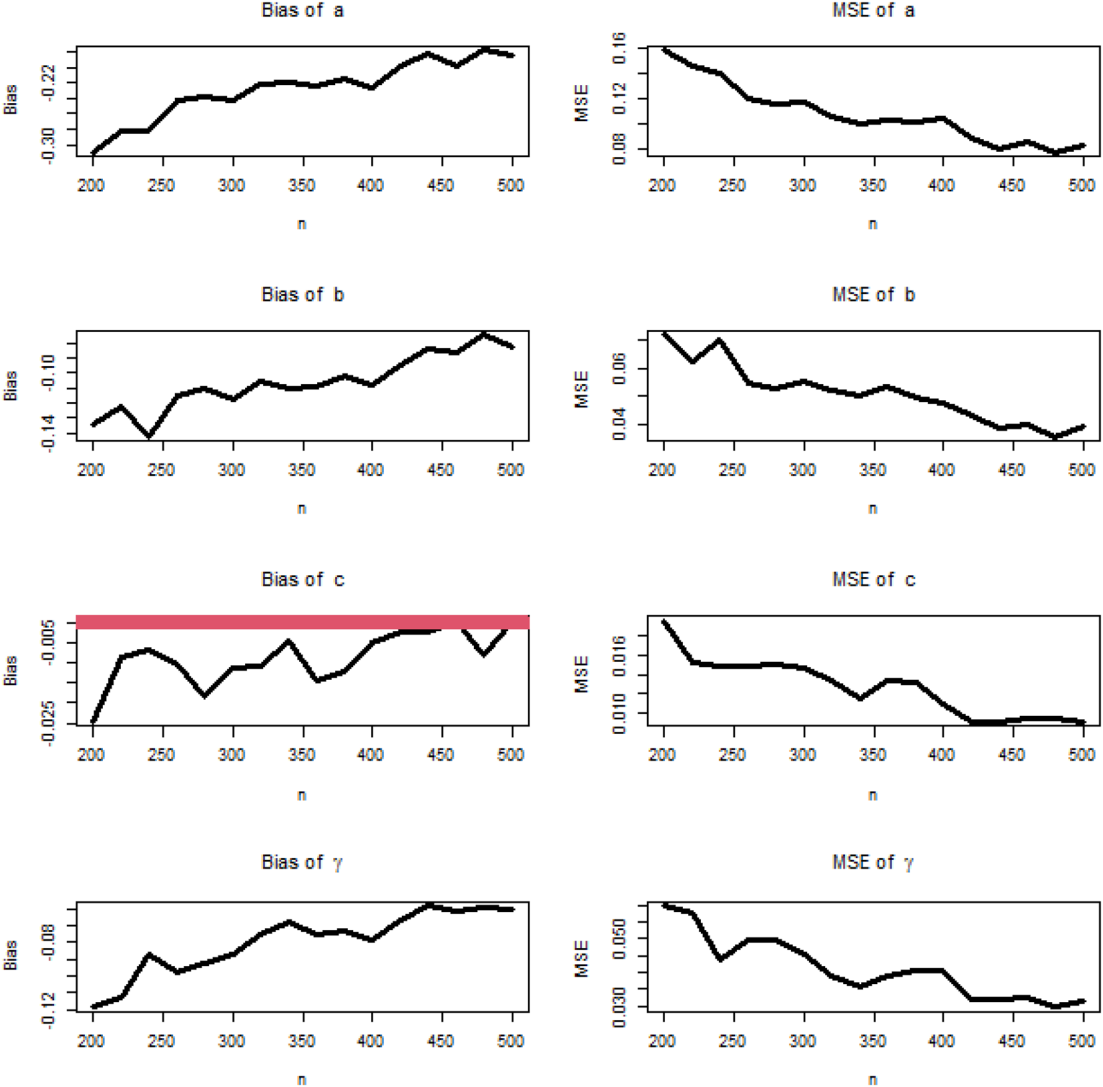
The results of the graphical simulations.

Figure 2 gives all simulations plots. The first row of Fig. 2 provides the biases and MSEs for parameter ${\mathcalligra{a}}\,$. The second row of Fig. 2 presents the biases and MSEs for parameter $\mathcalligra{b}\,\,$. The third row of Fig. 2 shows the biases and MSEs for parameter ${\mathcalligra{c}}\,$. The fourth row of Fig. 2 gives the biases and MSEs for parameter $\gamma$. Based on the first row of Fig. 2, the biases and MSEs get closer to zero as the sample size of the data generated increases. Based on the second row of Fig. 2, the biases and MSEs get closer to zero as the sample size of the data generated increases. Based on the third row of Fig. 2, the biases reached zero (see the red broken line) and the MSEs got closer to zero as the sample size of the data generated increases. Based on the fourth row of Fig. 2, the biases and MSEs get closer to zero as the sample size of the data generated increases. In general, we can say that the least squares method is considered an appropriate method for estimating the parameters of the new distribution, due to the important statistical properties that the estimators of this method have.

## Data modeling

Within the fields of statistics and mathematical modeling, a fundamental undertaking involves the comparison and evaluation of probability distributions. The aforementioned approach holds considerable importance in augmenting our comprehension of the process of real-life data collecting and enables researchers to derive useful inferences regarding the features of a community. However, the challenge of picking the most appropriate probability distribution can be quite hard, particularly when there are multiple different distributions available for a given dataset. In the present setting, the significance of actual data modeling is of utmost importance and should not be underestimated.

The assessment of the appropriateness of various probability distributions in relation to empirical datasets is a basic component of data modeling in practical contexts. This procedure serves various objectives.

Real-life (life-time) data modeling allows us to assess the appropriateness of several probability models for a given real dataset. It is imperative to acknowledge that a singular probability distribution may not sufficiently encompass all datasets. Data frequently demonstrates intricate patterns that necessitate a distribution that is either more advanced or adaptable. The utilization of real data modeling enables researchers to investigate a diverse array of probability distributions in order to ascertain the most accurate representation of the process by which the data is generated. After the identification of the ideal distribution, researchers have the ability to estimate its parameters and draw conclusions regarding the features of the population. The implications of these findings extend to the domains of data processing, hypothesis testing, and the forecasting of future observations. The utilization of real data modeling offers a method to evaluate the degree of concordance between the fitted distribution and the actual observed data. Goodness-of-fit tests are utilized to assess the extent to which the distribution that has been fitted aligns with the data that has been observed. A well-fitting distribution is shown by a low goodness-of-fit test value, which indicates a high degree of agreement between the distribution and the observed data.

In essence, the incorporation of actual data modeling holds significant importance within the realm of statistical analysis due to various compelling factors. This tool allows for the assessment of multiple probability distributions for a given dataset, aids in making inferences about the population, and supports the evaluation of the goodness-of-fit between a distribution and the observed data. This methodology is essential for making well-informed judgments and deriving significant insights from data across diverse domains and circumstances.

In this section of the manuscript, we elucidate two practical applications utilizing authentic data sets to underscore the utility and versatility of the GPWLO model. These applications utilize authentic data. The present study assesses the adequacy of the novel model in relation to many established rival models (refer to Table 2) in order to determine their relative performance. Table 2 presents a complete comparison across different families, encompassing:

I. The present study focuses on the Special Generalized Mixture-G (SGMX-G) family, hereafter referred to as SGMX.
II. The family Kumaraswamy (KUM-G).
III. The Log-logistic (OLL-G) class.
IV. The family of McDonald-G (Mc-G).
V. The Quasi log-logistic-G family, often known as the odd-G family, is denoted as QOLL-G.
VI. The exponentiated-G (EXP-G) family is a statistical distribution family.
VII. The seventh family under consideration is the transmuted Topp-Leone-G (TTL-G) family.
VIII. The family known as quasi transmuted topp-leone-G (QTTL-G) is the focus of this study.
IX. The Gamma-G (Gam-G) family is the subject of discussion in this section.
X. The Quasi family of the Burr-Hatke-G.
XI. The beta-G family.
XII. The proportional-reversed hazard-rate-G family.

**Table 2:** The competitive model.

Model	Abbreviation; Author
SGMX-Lomax	SGMXLO; Altun et al.^25^
Gamma-Lomax	Gam-LO; Cordeiro et al.^26^
TTL-Lomax	TTLLO; Yousof et al.^27^
Exp-Lomax	ExpLO; Gupta et al.^28^
QBH-Lomax	QBHLO; Yousof et al.^29^
OLL-Lomax	OLLLO; Chesneau and Yousof^30^
QTTL-Lomax	QTTLLO; Yousof et al.^27^
Kum-Lomax	KULO; Lemonte and Cordeiro^31^
QOLL-Lomax	QOLLLO; Chesneau and Yousof^30^
Lomax	LO; Lomax^32^
PRHR-Lomax	PRHRLO; –

This comparison entails evaluating the appropriateness of various families for modeling and analyzing particular datasets, taking into account criteria such as the quality of fit, estimation of parameters, and other statistical characteristics. Comparisons of this nature hold significant value in the process of picking the distribution family that is most suitable for a certain dataset or research challenge.

### The 1st data: survival times (84 aircraft windshield)

Survival data is of paramount importance in the field of actuarial science, as it serves as a fundamental factor in assessing the risks associated with insurance policies. Survival data provides critical information about the time-to-event for various occurrences, such as death, disability, or the termination of an insurance policy, among other scenarios. In actuarial science, survival data is applied in modeling various types of risks, including mortality, morbidity, and longevity. It also plays a crucial role in estimating the future value of potential losses. This information is instrumental in the development of actuarial tables, which, in turn, are used to calculate insurance premiums, establish reserves, and design insurance products. Survival data is also employed to assess the impact of various individual characteristics, such as age, gender, health status, and lifestyle choices, on the likelihood of a specific event occurring. This knowledge is integral to the creation of underwriting guidelines and informed decisions regarding policy approval and pricing. In summary, survival data forms an essential component of actuarial science and is vital for evaluating the risks associated with insurance policies. Ensuring the accuracy and quality of this data is crucial for precise actuarial modeling and projections, allowing insurance companies to effectively manage their risks and financial obligations. The mention of the 1st real-life data set representing failure times of 84 aircraft windshields from Murthy et al.^33^ likely suggests the application of survival data in analyzing and modeling the reliability and failure patterns in this specific context. For more real applications, data and related competitive models, see Altun et al.^34^, Goual et al.^35^, Ibrahim^36^, Ibrahim and Yousof^37^, Mansour et al.^38^ and Yadav et al.^39^.

### The 2nd data: service times (63 aircraft windshield)

Queuing theory is a mathematical framework used to model systems with waiting lines, such as contact centers, traffic flow, and manufacturing processes. It involves modeling the arrival of customers and their service times using probability distributions. Stochastic properties are used to determine average waiting times and queue sizes in these systems. The mention of the 2nd real-life data set representing service times of 63 aircraft windshields from various sources suggests the application of queuing theory in analyzing and modeling service times in this context. Queuing theory can help in understanding and optimizing processes that involve waiting times, such as service queues. In practical applications of data analysis, various graphical tools are often used to visualize and analyze data, including:

I. Quantitative-quantile plot (Q–Q plot).
II. Kernel density estimation (KDE) plot.
III. Total time in test (TTT) plot.
IV. Box plot.

**Figure 3:**
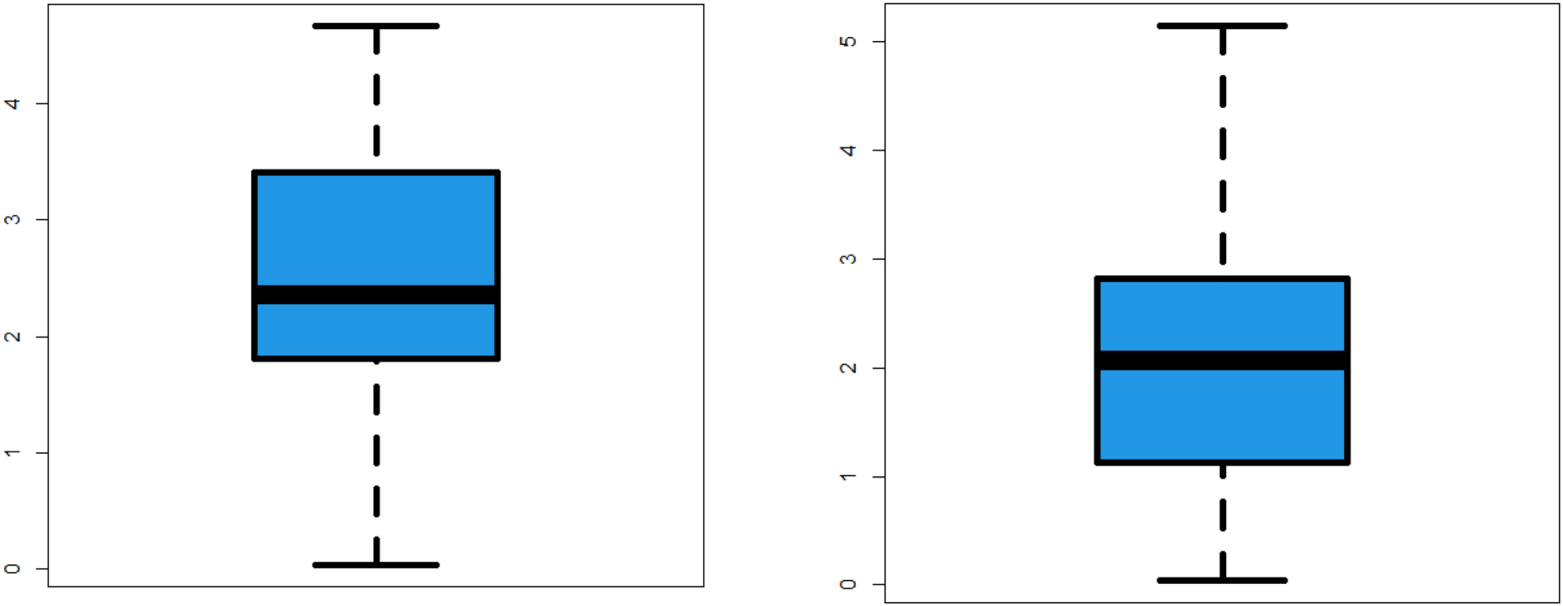
Box plot for the 1st data set (left/first plot) and for 2nd data set (right/second plot).

**Figure 4:**
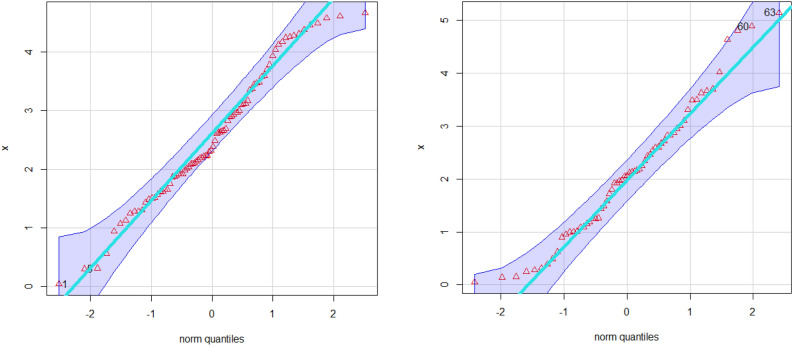
Q–Q plot for the 1st data set (left/first plot) and for 2nd data set (right/second plot).

**Figure 5:**
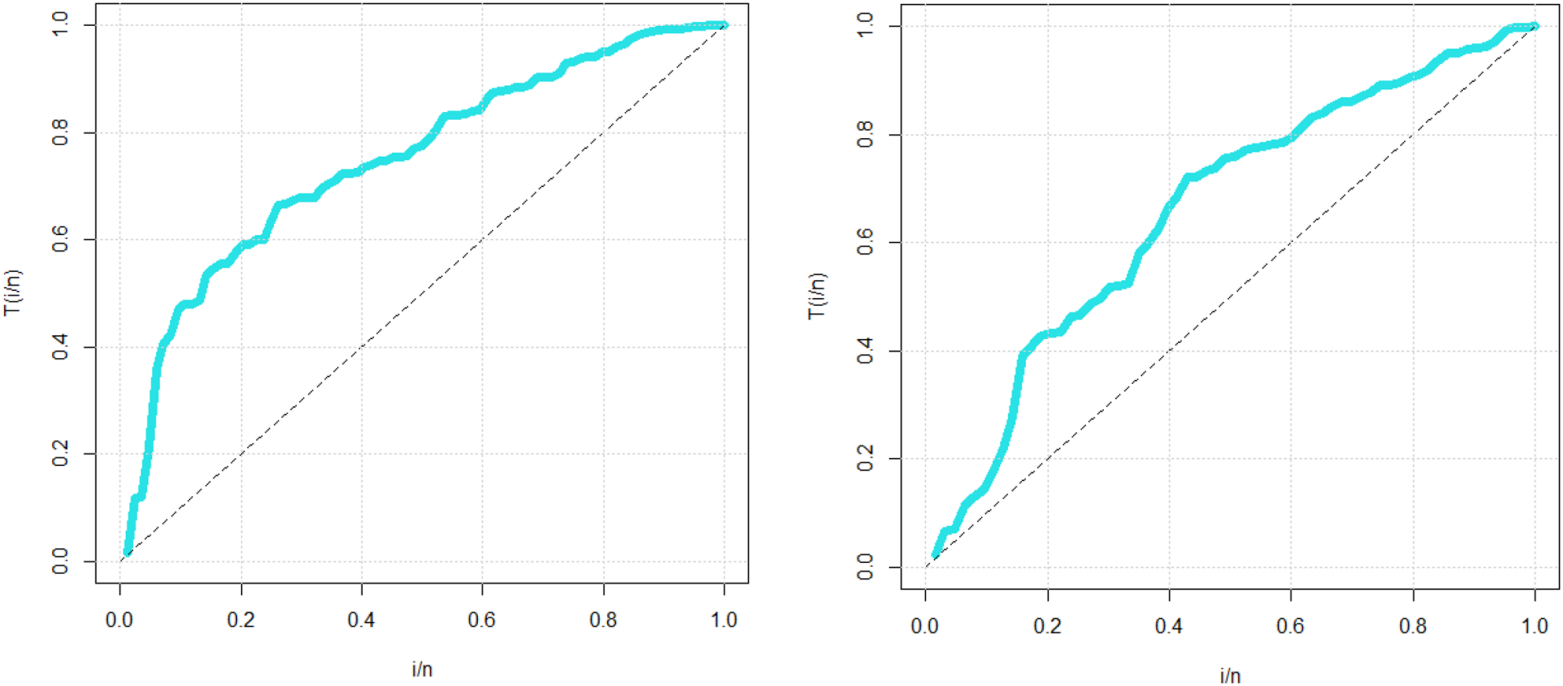
TTT plot for the 1st data set (left/first plot) and for 2nd data set (right/second plot).

**Figure 6:**
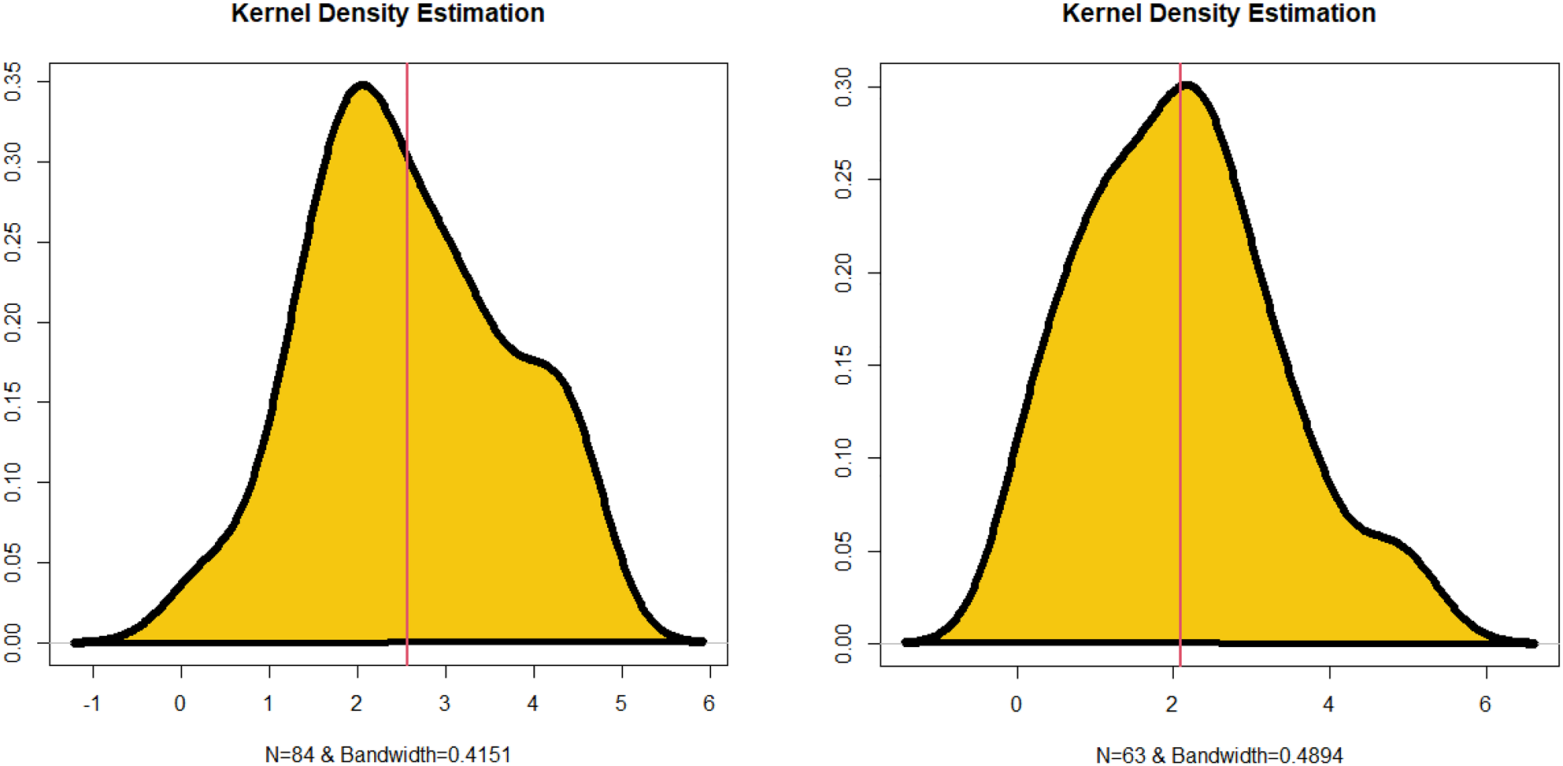
Nonparametric-KDE for the 1st data set (left/first plot) and for 2nd data set (right/second plot).

These graphical tools aid in the exploration, visualization, and interpretation of real-life data sets, making them valuable in various fields of research and analysis. As can be seen in Fig. 3, there were no anomalous findings or outliers that were found. Figure 4 illustrates the “Q–Q plot” that is utilized while doing the “normality” test. When we take a look at Fig. 4, we can see that the phenomenon known as “normality” is almost entirely present. The plot labeled “total time test (TTT)” has been supplied so that an investigation into the form of the empirical HRF can be carried out (refer to Fig. 5). Figure 5 indicates quite clearly that both sets of data have a pattern that can be described as “asymmetric monotonically increasing” for the HRF. The Kernel Density Estimation (KDE), which can be located in Fig. 6, is a tool that may be utilized in a method that does not require the usage of parameters in order to study the initial form of real data. Figures 5 and 6 show the estimated PDF (EPDF) and estimated HRF (EHRF), respectively, for the two distinct data sets that were used in the study.

The goodness-of-fit (GOF) statistics given below are considered and used to evaluate and compare the competing models:

I. Akaike Information Criterion (AK-IC).
II. Bayes-IC (BY-IC).
III. Consistenting-AK-IC (C-AKIC).
IV. Hannan and Quinn-IC (HQN-IC).

Estimations can be found in Table 3 under the heading “MLEs”, while the value of all specified GOF statistics can be found in Table 4. This data pertains to the failure times, which can be found listed in their full in Tables 3 and 4. This information is relevant to those tables. Tables 5 and 6 include the relevant results for the times of service data. Table 5 has the estimations and the accompanying SE, and Table 6 contains the value of the GOFs statistics.

**Table 3:** The results of estimation process (MLEs and SEs) for the 1st data under all competitive models.

Computing model	Estimates under the MLE method and their estimation errors			
GPWLO(${\mathcalligra{a}}\,,\mathcalligra{b}\,\,,{\mathcalligra{c}}\,,\gamma$)	1.908789	0.2559808	6.644979	0.155347
	(0.878817)	(0.00877)	(0.09911)	(0.003214)
KULO(${\mathcalligra{a}}\,,\mathcalligra{b}\,\,,{\mathcalligra{c}}\,,\gamma$)	2.61599	100.2765	5.27754	78.6778
	(0.3854)	(120.185)	(9.8155)	(186.001)
TTLLO(${\mathcalligra{a}}\,,\mathcalligra{b}\,\,,{\mathcalligra{c}}\,,\gamma$)	− 0.80754	2.476768	(156,910)	(386,243)
	(0.13911)	(0.54854)	(1601.01)	(120.083)
TTLLO(${\mathcalligra{a}}\,,\mathcalligra{b}\,\,,{\mathcalligra{c}}\,,\gamma$)	3.60346	33.63001	4.83349	117.8398
	(0.61999)	(63.7443)	(9.23876)	(420.093)
PRHRLO(${\mathcalligra{a}}\,,\mathcalligra{b}\,\,,{\mathcalligra{c}}\,$)	3.729 × 10^6^	4.723 × 10^−1^	4.511 × 10^6^	
	1.197 × 10^6^	(0.00014)	(37.14991)	
SGMXLO(${\mathcalligra{a}}\,,\mathcalligra{b}\,\,,{\mathcalligra{c}}\,$)	− 1.057 × 10^−1^	9.167 × 10^6^	1.209 × 10^7^	
	(0.12769)	(48,986.5)	(512.234)	
QTTLLO(${\mathcalligra{a}}\,,\mathcalligra{b}\,\,,{\mathcalligra{c}}\,$)	− 0.847653	5.52057	1.156786	
	(0.100143)	(1.18880)	(0.09566)	
OLLLO(${\mathcalligra{a}}\,,\mathcalligra{b}\,\,,{\mathcalligra{c}}\,$)	2.3260625	(7.243 × 10^5^)	2.347 × 10^6^)	
	(2.146 × 10^−1^)	(1.963 × 10^4^)	(2.609 × 10^1^)	
ExpLO(${\mathcalligra{a}}\,,\mathcalligra{b}\,\,,{\mathcalligra{c}}\,$)	3.626659	20,074.99	26,256.79	
	(0.62881)	(2041.809)	(99.4363)	
Gam-LO(${\mathcalligra{a}}\,,\mathcalligra{b}\,\,,{\mathcalligra{c}}\,$)	3.587551	52,191.499	37,028.93	
	(0.513778)	(79,558.19)	(81.1665)	
QOLLLO(${\mathcalligra{a}}\,,\mathcalligra{b}\,\,$)	3.8900821	0.573777		
	(0.36566)	(0.016870)		
QBHLO(${\mathcalligra{a}}\,,\mathcalligra{b}\,\,$)	10,801,709	51,367,189.98		
	(983,308.29)	(23,231.992)		
LO(${\mathcalligra{a}}\,,\mathcalligra{b}\,\,$)	51,425.433	131,700.324		
	(5933.555)	(296.1265)

**Table 4:** GOFs statistics for all competitive models under the 1st data.

Model	− ℓ	AK-IC	C-AKIC	BY-IC	HQN-IC
GPWLO	126.8381	261.0430	261.7220	271.2122	265.9021
OLLLO	136.4083	274.8916	275.948	283.1014	278.7782
PRHRPa	162.8572	331.7932	332.9537	339.0033	333.9918
TTLLO	136.5749	279.1429	279.6730	288.8544	283.1173
Gam-LO	136.9991	282.8129	282.9919	290.1984	286.7535
TTLLO	138.7919	285.4818	286.1911	295.2305	288.3111
ExpLO	142.1480	288.7910	288.0019	296.1119	292.7554
QBHLO	168.6145	342.2001	344.1171	345.0703	346.0015
QOLLLO	142.8910	289.8391	289.2328	295.5304	292.6342
QTTLLO	153.7319	313.9515	314.2828	321.2542	317.0841
SGMXLO	143.0819	292.1719	294.0029	299.4544	294.1651
LO	164.9981	333.1971	338.0004	339.8601	336.0442

**Table 5:** The results of estimation process (MLEs and SEs) for the 2nd data under all competitive models.

Computing model	Estimates under the MLE method and their estimation errors			
GPWLO(${\mathcalligra{a}}\,,\mathcalligra{b}\,\,,{\mathcalligra{c}}\,,\mathcalligra{b}\,\,$)	− 1.045424	0.854304	1.12329	0.314593
	(1.804277)	(0.50044)	(0.51982)	(0.13009)
TTLLO(${\mathcalligra{a}}\,,\mathcalligra{b}\,\,,{\mathcalligra{c}}\,,\gamma$)	1.924435	31.25944	4.966661	165.574
	(0.3165)	(316.554)	(50.5670)	(337.21)
KULO(${\mathcalligra{a}}\,,\mathcalligra{b}\,\,,{\mathcalligra{c}}\,,\gamma$)	1.669874	60.5676	2.56477	65.0649
	(0.25744)	(88.054)	(4.75833)	(177.984)
TTLLO(${\mathcalligra{a}}\,,\mathcalligra{b}\,\,,{\mathcalligra{c}}\,,\gamma$)	− 0.60445	1.7785W	2123.391	4821.792
	(0.21909)	(0.41565)	(163.914)	(201.02)
QTTLLO(${\mathcalligra{a}}\,,\mathcalligra{b}\,\,,{\mathcalligra{c}}\,$)	− 0.671756	2.744999	1.012344	
	(0.187111)	(0.66954)	(0.114122)	
PRHRPa(${\mathcalligra{a}}\,,\mathcalligra{b}\,\,,{\mathcalligra{c}}\,$)	1.5980 × 10^6^	3.955 × 10^−1^	1.322 × 10^6^	
	2.0450 × 10^3^	0.0123 × 10^−1^	0.933 × 10^6^	
SGMXLO(${\mathcalligra{a}}\,,\mathcalligra{b}\,\,,{\mathcalligra{c}}\,$)	− 1.0405 × 10^−1^	6.459 × 10^6^	6.397 × 10^6^	
	(4. 36 × 10^−10^)	(3.298 × 10^6^)	(3.89834)	
Gam-LO(${\mathcalligra{a}}\,,\mathcalligra{b}\,\,,{\mathcalligra{c}}\,$)	1.907345	35,848.411	39,197.505	
	(0.32443)	(6945.039)	(151.6522)	
OLLLO(${\mathcalligra{a}}\,,\mathcalligra{b}\,\,,{\mathcalligra{c}}\,$)	1.669814	6.349 × 10^5^	2.0144 × 10^6^	
	(1.111 × 10^−1^)	(1.611 × 10^4^)	7.2350 × 10^6^	
ExpLO(${\mathcalligra{a}}\,,\mathcalligra{b}\,\,,{\mathcalligra{c}}\,$)	1.987945	22,933.150	32,887.855	
	(0.34876)	(3201.511)	(162.2555)	
QBHLO(${\mathcalligra{a}}\,,\mathcalligra{b}\,\,$)	140,555.454	532,034.343		
	(422.0154)	(28.52431)		
QOLLLO(${\mathcalligra{a}}\,,\mathcalligra{b}\,\,$)	2.372366	0.6911129		
	(0.268398)	(0.04499)		
LO(${\mathcalligra{a}}\,,\mathcalligra{b}\,\,$)	99,263.836	207,019.01		
	(11,860.56)	(301.2679)

**Table 6:** GOFs statistics for all competitive models under the 2nd data.

Model	− ℓ	AK-IC	C-AKIC	BY-IC	HQN-IC
GPWLO	98.14441	204.1213	204.7872	212.5722	207.64322
QBHLO	112.6253	229.2293	229.4492	233.4053	230.8665
KULO	100.8215	209.7583	210.4253	218.3159	213.1989
QTTLLO	112.1916	230.3515	230.7918	236.8905	232.9983
TTLLO	102.4462	212.9718	213.5823	221.4561	216.2544
ExpLO	103.5511	213.0783	213.1541	219.5345	215.6597
Gam-LO	102.8293	211.6900	212.0917	218.0992	214.1908
PRHRPa	109.3994	224.5994	225.9119	231.4072	227.3936
SGMXLO	102.8996	211.7880	212.9941	218.2405	214.3439
TTLLO	102.9941	213.9943	214.0992	222.4913	217.2908
OLLLO	104.9919	215.8388	216.2291	222.2525	218.3501
QOLLLO	110.7137	225.4256	225.6499	229.7405	227.3333
LO	109.1276	222.5198	222.0896	226.8055	224.1298

Based on Table 4, the GPWLO model provided the best fits with − ℓ = 126.838, AK-IC = 261.043, C-AKIC = 261.707, BY-IC = 271.212 and HQN-IC = 265.902.

**Figure 7:**
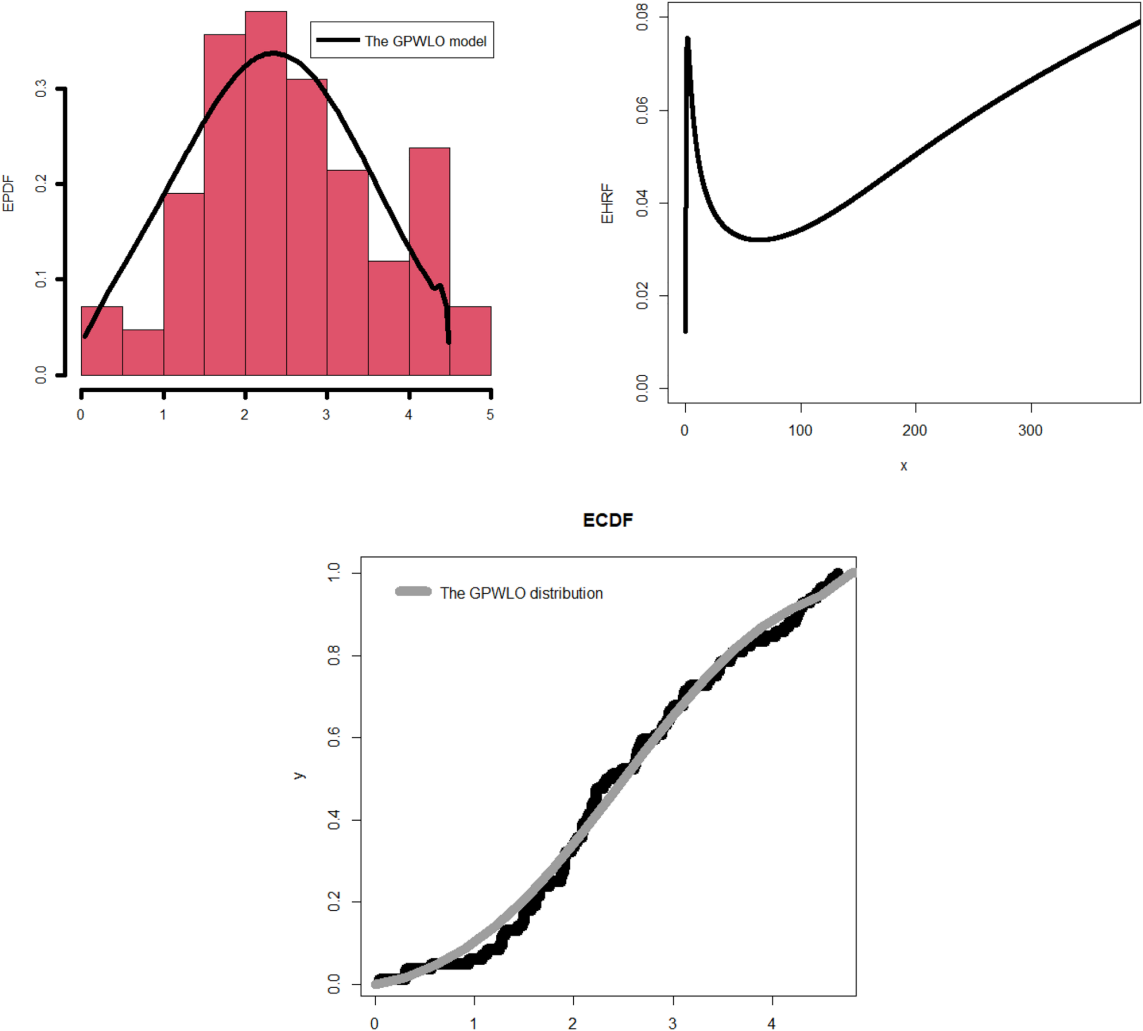
EPDF (left/first graph), the EHRF (right/second graph) and ECDF (last/third plot) for the 1st data.

Due to Fig. 7, the new GPWLO model gives adequate results, and indicates that it is indeed the most appropriate model among all models for the 1st data, according to the EPDF (see left graph of Fig. 7), the EHRF (see right graph of Fig. 7) and ECDF (see last plot of Fig. 7).

Based on Table 6, the GPWLO model provided the best fits with − ℓ = 98.1444, AK-IC = 204.121, C-AKIC = 204.788, BY-IC = 212.572 and HQN-IC = 207.6432.

**Figure 8:**
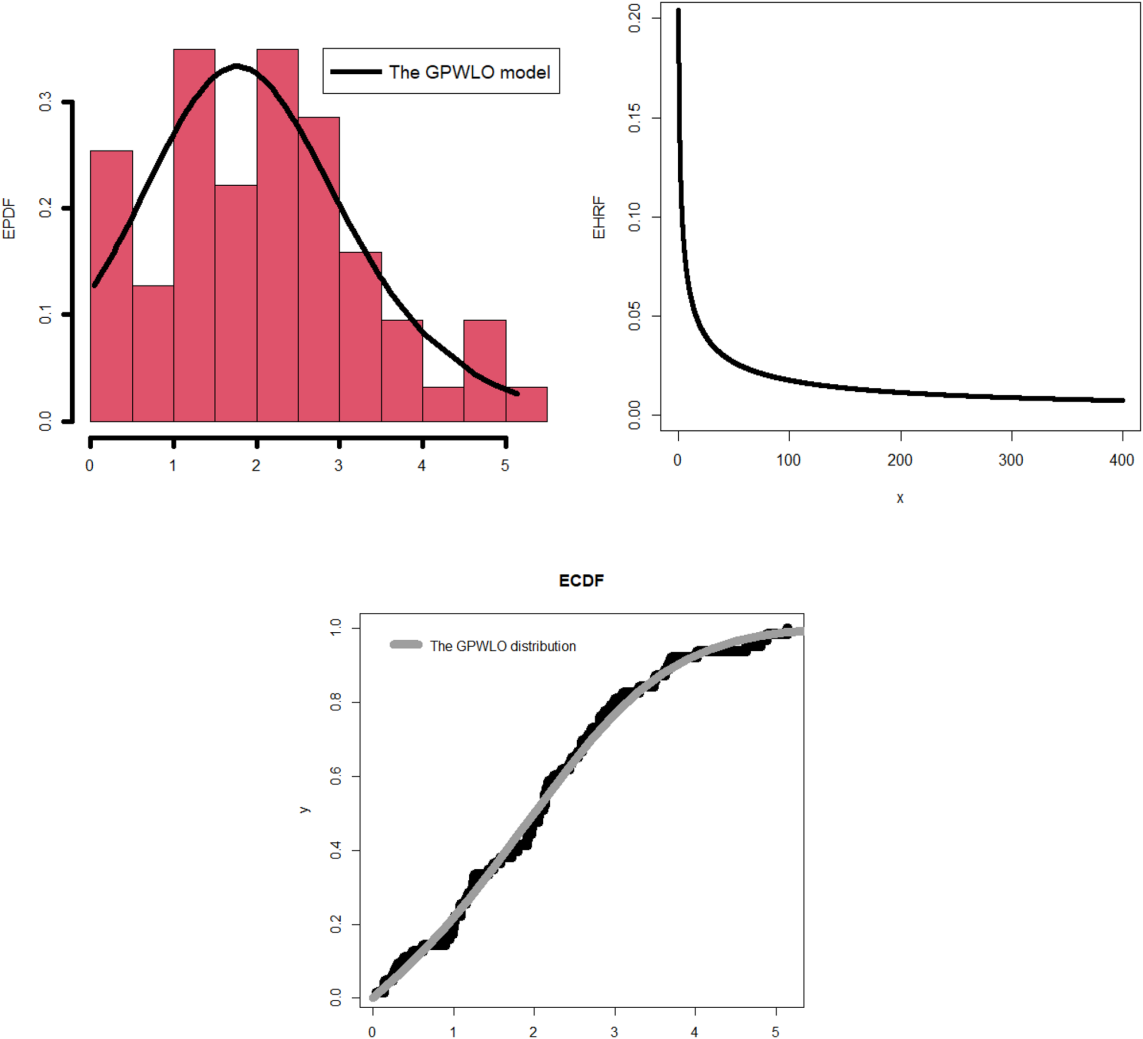
EPDF (left/first graph), EHRF (right/second graph) and ECDF (last/third plot) for the 2nd data.

Due to Fig. 8, the new GPWLO model gives adequate results, and indicates that it is indeed the most appropriate model among all models for the 2nd data, according to the EPDF (see left graph of Fig. 8), the EHRF (see right graph of Fig. 8) and ECDF (see last plot of Fig. 8). When compared to the other fitted models, the GPWLO model produces the lowest values for the AK-IC, C-AKIC, BY-IC, and HQN-IC. This is evidenced by the data that is presented in Tables 4 and 6. After taking all the aforementioned considerations into account, it is feasible that this model will end up being selected as the best available alternative. In Fig. 7, the EPDF can be found on the left graph, the EHRF can be found on the right graph, and the ECDF can be found on the last plot for the first data. For the second set of data, Fig. 8 presents the EPDF (shown in the left graph), the EHRF (shown in the right graph), and the ECDF (shown in the last plot).

## Conclusions

The topic of investigation for the research paper that you have outlined is compound families of probability distributions with copulas, with an emphasis on the mathematical underpinnings of these distributions as well as their practical applications in real life datasets. These compound families, which are frequently constructed on copulas, provide a framework that is both flexible and versatile for modeling a broad variety of phenomena, such as the amount of time it takes for a certain system to fail, the amount of money lost, the number of accidents that occur, and more. They come in especially handy in situations in which an accurate representation of the relationships between a number of different variables is required. The study also presents a novel compound family that has been dubbed the “Generated Poisson Weibull G family”. This family is comprised of components that are taken from both the Poisson (under trancation) the generalized Weibull G statistical families. This innovative family of distributions offers a variety of statistical properties, and the research investigates a number of different approaches to derive these features. Additionally, the research extends to the production of novel bivariate G families by applying various copulas, such as copula under CTN, copula under Renyi concept and Ali, Mikhail, Haq copula, among many others relevant copulas. This is accomplished by the use of diverse copulas. The modeling of dependencies between two or more random variables is made possible by the use of these copulas. In addition, the Lomax model with a single parameter is mentioned in the study. This model is a significant focal point of interest. In addition, the relevance and application of the novel compound family and copula-based models are demonstrated with the help of two instances taken from real life situations. Overall, the findings of this study contribute to the understanding of compound families of probability distributions with copulas and their application in a variety of domains. These findings provide insights into the mathematical foundations of these distributions as well as their practical applications in the modeling of complicated real life occurrences.

The study of compound families is a fruitful area of research, particularly for professionals and experts working in the domains of mathematics and statistical modeling. In this particular setting, we will provide a rundown of some potential future points that might be of assistance to the academics in conducting additional statistical research. The following points are potential future considerations:

I. The utilization of copulas facilitates the examination of newly formed compound families. It is imperative for researchers to undertake a comprehensive investigation into the advancement of innovative copula functions and their integration with various marginal distributions. This pursuit is essential to enhance the adaptability of modeling techniques and effectively address the diverse range of dependencies noticed in real life datasets. These dependencies can be taken into consideration by means of modeling.
II. The application of compound families technique to the study of time series data. This study aims to explore the application of copulas and compound families in the modeling and prediction of multivariate time series. It specifically considers the temporal dependencies and serial correlations observed in real life datasets sourced from various domains such as finance, economics, and related fields.
III. This section aims to explore the difficulties that may arise when utilizing compound families with copulas in the context of high-dimensional data sets, along with the possible remedies that may be employed to address these obstacles. This study aims to explore the application of dimensionality reduction techniques, sparse copulas, and other strategies to address the issues posed by the curse of dimensionality and enhance the efficiency of the estimation process.
IV. In this study, we want to examine the resilience of compound families through the utilization of copulas, particularly in scenarios where outliers or model misspecification may arise. Performing a sensitivity analysis is necessary to evaluate the influence of different copula families and marginal distributions on the overall outcomes, as well as to determine the generalizability of the findings to the specific datasets under consideration.
V. The objective of this study is to investigate Bayesian approaches in the estimation of the overall count of compound families through the utilization of copulas. This inquiry delves into the potential application of Bayesian priors as a technique for integrating prior knowledge and uncertainties into the modeling process. This proposed framework offers a comprehensive and cohesive way to effectively manage intricate data structures.
VI. The application of compound families with copulas can be extended to datasets derived from new industries, including but not limited to Internet of Things (IoT) data, social network data, and cybersecurity data. This study aims to examine the methodologies via which these models can offer valuable perspectives on the associations and risk patterns present in these datasets. Additionally, it seeks to explore their ability to handle unique conditions.
VII. In this section, we will focus on the development of methodologies for evaluating and visually representing the outcomes of compound families through the utilization of copulas. Subsequently, we will go on to the subsequent stage. The seventh item. The appreciation and effective communication of the complex interconnections present in data sets are of utmost importance in practical domains such as risk management and decision-making.
VIII. In the context of compound families with copulas, the subsequent procedure entails examining the causal connections that are present among the variables. This represents a procedural stage. VIII. Further investigation is warranted to ascertain the potential of these models in enhancing the Granger causality analysis and the process of deriving causal conclusions from multivariate data sets.
IX. The objective of this study is to explore the representation of evolving dependencies over time through the utilization of compound families employing copulas. Additionally, this investigation aims to examine the applicability of this approach within the setting of changing conditions in the data. The objective of this study is to examine the manner in which changing dependencies over a period of time might be effectively represented by compound families utilizing copulas. In the context of analyzing non-stationary interactions in empirical data, it is imperative to conduct comprehensive study on two distinct methodologies: dynamic copulas and time-varying compound families.
X. The objective of this study is to examine the feasibility of incorporating compound families and copulas in order to create a model that can accurately represent interdependence characterized by both asymmetry and multimodality. Given the significance of understanding extreme events and tail risk across several domains, it is imperative to investigate the identification and modeling of tail dependencies. Therefore, it is imperative to do an investigation into the modeling of tail dependencies.
XI. Neutrosophic statistics serves as an extension of classical statistics, specifically employed in scenarios where the data under analysis originates from a complicated process or an environment characterized by uncertainty. In the endeavor to quantify different aspects of uncertainty, specific neutrosophic statistical methodologies may incorporate concepts such as measurements relating to probability. When elucidating and scrutinizing neutrosophic data, one may employ entropy or metrics associated with the concepts of possibility, necessity, and indeterminacy.
XII. The primary emphasis of philosophical statistics lies in addressing the challenges associated with complex and ambiguous data. The utilization of statistical methodologies adapted for neutrosophic sets is feasible; yet, it is important to note that these techniques may not align with the usual statistical analysis that relies on probability distributions.
XIII. The design of neutrosophic sets often incorporates elements from fuzzy set theory. Fuzzy sets assign different degrees of membership to elements inside a set. The truth, indeterminacy, and falsehood components of a neutrosophic set can be effectively represented through the utilization of fuzzy membership functions.
XIV. The potential for further advancement in neutrophilic statistics lies in the integration of probability theory with conventional statistical approaches, which would provide a comprehensive framework for handling complex, uncertain, and imprecise data. Researchers may develop methodologies that effectively combine neutrosophic sets with probability distributions in order to measure different aspects of uncertainty.
XV. The potential for further developments in neutrosophic statistics is anticipated to yield more advanced techniques for data analysis, incorporating the utilization of both neutrosophic sets and probability distributions. This has the potential to facilitate more precise and resilient modeling and analysis of data derived from ambiguous environments, such as those commonly seen in domains like finance, healthcare, and environmental research.

## Data Availability

The data will be provided by Haitham M. Yousof upon request.
